# *FoxA4* Favours Notochord Formation by Inhibiting Contiguous Mesodermal Fates and Restricts Anterior Neural Development in *Xenopus* Embryos

**DOI:** 10.1371/journal.pone.0110559

**Published:** 2014-10-24

**Authors:** Sabrina Murgan, Aitana Manuela Castro Colabianchi, Renato José Monti, Laura Elena Boyadjián López, Cecilia E. Aguirre, Ernesto González Stivala, Andrés E. Carrasco, Silvia L. López

**Affiliations:** Laboratorio de Embriología Molecular “Prof. Dr. Andrés E. Carrasco,” Instituto de Biología Celular y Neurociencia, Facultad de Medicina, Universidad de Buenos Aires, Ciudad Autónoma de Buenos Aires, Argentina; Instituto Gulbenkian de Ciência, Portugal

## Abstract

In vertebrates, the embryonic dorsal midline is a crucial signalling centre that patterns the surrounding tissues during development. Members of the FoxA subfamily of transcription factors are expressed in the structures that compose this centre. *Foxa2* is essential for dorsal midline development in mammals, since knock-out mouse embryos lack a definitive node, notochord and floor plate. The related gene *foxA4* is only present in amphibians. Expression begins in the blastula –*chordin* and –*noggin* expressing centre (BCNE) and is later restricted to the dorsal midline derivatives of the Spemann's organiser. It was suggested that the early functions of mammalian *foxa2* are carried out by *foxA4* in frogs, but functional experiments were needed to test this hypothesis. Here, we show that some important dorsal midline functions of mammalian *foxa2* are exerted by *foxA4* in *Xenopus*. We provide new evidence that the latter prevents the respecification of dorsal midline precursors towards contiguous fates, inhibiting prechordal and paraxial mesoderm development in favour of the notochord. In addition, we show that *foxA4* is required for the correct regionalisation and maintenance of the central nervous system. *FoxA4* participates in constraining the prospective rostral forebrain territory during neural specification and is necessary for the correct segregation of the most anterior ectodermal derivatives, such as the cement gland and the pituitary anlagen. Moreover, the early expression of *foxA4* in the BCNE (which contains precursors of the whole forebrain and most of the midbrain and hindbrain) is directly required to restrict anterior neural development.

## Introduction

The dorsal midline (DML) of vertebrate embryos is an important signalling centre for the development of the surrounding tissues. Signals emanating from this centre are involved in the specification of ventral neural fates and sclerotome, in proliferation and survival of neural precursors, in axonal pathfinding, and in the patterning of the axial vasculature [1] [2] [3] [4] [5] [6] [7].

The anterior DML is composed by the prechordal mesoderm and the anterior part of the floor plate (FP). Three structures intimately juxtaposed constitute the posterior DML: the posterior part of the FP, the notochord, and the dorsal midline of the endoderm (in amniotes) or the hypochord (in anamniotes). The FP occupies the ventral midline of the neural tube, and its posterior part comprises the medial FP (MFP) and the lateral FP (LFP) [8] [9] [10]. Among the DML components, the prechordal mesoderm (PM), the hypochord, the notochord and the MFP are descendants of the vertebrate organiser, while the anterior FP and the LFP are neural ectoderm derivatives induced by the organiser-derived DML structures [11] [12].

The vertebrate organiser and its DML derivatives express *foxA* genes, which belong to a family of transcription factors containing a DNA binding domain known as “winged helix” [13] [14] [15] [16]. The first member of the family, *fork head* (*fkh*), was isolated from *Drosophila melanogaster* [17] [18]. *Fox* genes were further found in a wide variety of organisms, ranging from yeast to human. They are involved in a broad spectrum of cellular and developmental processes, including specification of germ layers, cell-cycle regulation, metabolism, and organogenesis [19] [20] [21] [22].

In mouse embryos, *foxa2* is expressed at late streak stage in the node (the mammal equivalent of the amphibian organiser), and slightly later, in the node descendants composing the axial tissues, i.e. the notochord, the prechordal mesoderm, the FP and the midline of the underlying endoderm [15] [23]. *Foxa2* is essential for DML development in mammals, since knock-out mouse embryos lack a definitive node, notochord and FP. One of the conclusions of these works was that neural induction can occur even in the absence of DML signalling. However, the dorso-ventral (D–V) pattern of the neural tube was abnormal and the anterior head structures were truncated or reduced [24] [25] [26] [27] [28].

In *Xenopus*, *foxA2* is weakly expressed in the organiser, while its related gene *foxA4* is strongly expressed in this region. At early neurulae, *foxA2* is only expressed by FP cells, while *foxA4* is expressed in all organiser derivatives in the DML [29] [30] [31] [32]. Therefore, at least until the closure of the neural tube, the expression pattern of *Xenopus foxA4* resembles more faithfully that of mouse *foxa2*. This suggested that the early functions of mouse *foxa2* are carried out by *foxA4* in frogs [32]. However, functional experiments are needed to demonstrate whether *foxA4* is the functional equivalent of mammalian *foxa2*. So far, orthologues for *foxA4* were only found in amphibians [21].

Early gain-of-function studies indicated that *foxA4* has the ability to participate during D–V patterning of the neural tube and mesoderm. Injection of *foxA4* mRNA repressed the differentiation of dorsal neurons in the spinal cord [31]. The expression of the FP marker *F-spondin* was ventrally expanded or ectopically induced in the dorsal neural tube after *foxA4* overexpression, although both results appeared with low frequency and were restricted to the hindbrain region. This suggested that *foxA4* might play a role in FP development [33]. However, to confirm the normal function of *foxA4* during *Xenopus* development, loss-of-function studies were required.

The first attempts to inhibit *foxA4* function in *Xenopus* employed recombinant proteins comprising the Fox DNA-binding domain fused with the *Drosophila* engrailed transcriptional repressor domain [34]. *FoxA4-EnR* mRNA-injected tadpoles exhibited severe anterior and posterior truncations, and often contained a shortened notochord which was either split or thickened, most likely due to the disruption of convergent extension movements during gastrulation [34]. However, there exists controversy about the transcriptional behaviour of FoxA4, since earlier studies proposed that it functions as a transcriptional activator [34] [35], while a more recent report claims that it acts as a transcriptional repressor on the anterior gene *Xanf1* [36]. Thus, the conclusions obtained with recombinant proteins harbouring heterologous activator or repressor domains would depend on the transcriptional behaviour of the protein under study in a particular context, if this protein displays dual and opposite transcriptional functions. By using a different strategy with an antisense morpholino oligonucleotide (MO) to knock-down *foxA4* in *Xenopus laevis*, it was shown that this gene controls the posterior medial limit of *Xanf1*, thus restricting its caudal expansion within the DML, as expected from the known expression of *foxA4* in the FP [36]. However, the confinement of *foxA4* expression to the DML structures just begins during gastrulation. At earlier stages this gene is transcribed in the **b**lastula –***c*** *hordin* and –***n*** *oggin* **e**xpressing centre (BCNE), which is composed by precursors of the Spemann's organiser and of the whole forebrain and most of the midbrain and hindbrain [37] [38] [39]. In this context, to better understand the normal requirement of *foxA4*, we used the same MO as Martynova et al. (2004) [36] but delivered in such way to affect the whole descendants of the BCNE. With this approach, we obtained more profound effects on the central nervous system (CNS), compromising the whole anterior neural plate. Our results indicate that *foxA4* is required for the normal anterior-posterior (A–P) pattern of the CNS, constraining the prospective rostral forebrain territory during neural specification, and for the segregation of the most anterior ectodermal derivatives, such as the cement gland and the pituitary anlagen. The early expression of *foxa4* in the BCNE is directly involved in restricting anterior neural development. In addition, we provide new evidence that this gene prevents the respecification of DML precursors towards contiguous fates, favouring notochord development at the expense of paraxial mesoderm or more anterior axial fates (prechordal mesoderm).

## Materials and Methods

### Embryo culturing, manipulations, morpholinos, cDNA cloning and processing

All animal experiments in this report followed the rules recommended by the Institutional Review Board for the Care and Use of Laboratory Animals (CICUAL) in the School of Medicine, University of Buenos Aires, Argentina, which approved this study. Wild-type and albino *Xenopus laevis* embryos were obtained using standard methods and staged according to Nieuwkoop and Faber [40].

We employed the previously described FoxA4MO [36], which recognizes the 5′UTR of *foxA4a* and its homeologue *foxA4b*. As control morpholino (CtrlMO), we used the standard control oligonucleotide or the random control oligonucleotide 25-N (Gene Tools, LLC, OR, USA). Except for the latter, all MOs were modified with 3′-fluorescein.

The coding sequence of *foxA4a* cDNA (*foxA4CDS*) was amplified by PCR with Platinum Pfx DNA Polymerase (Invitrogen). We used the full length *foxA4a* cDNA as template (GenBank: X65171.1) [31] and the following pair of primers: FoxA4a forward: 5′-ATGCTAAATAGAGTCAAACT-3′; FoxA4a reverse: 5′-TTAAAGGGAGCTGAGGATAG-3′. The amplified product was inserted into the EcoRV site of pT7TS with T4 DNA ligase (Invitrogen), and the resulting construct was linearized with EcoRI and transcribed with T7 RNA polymerase. Synthetic capped mRNA was obtained as described [41]. For overexpression studies, we used either the full length *foxA4a* mRNA (*foxA4FL*) [31] or the *foxA4a* mRNA which only contains the coding sequence (*foxA4CDS*). This mRNA lacks the target sequence of FoxA4MO and was used for rescue experiments. To block mesoderm induction, we injected synthetic capped cerberus-short mRNA (Cer-S) [42] [37].

All MOs and *foxA4* mRNA injections were directed towards the animal region. In some experiments, unilateral injections were performed into one cell at the 2 or 4-cell stage, to compare changes in relation to the non-injected side. In other experiments, in order to attain a bilateral homogenous distribution of the molecules, injections were performed at 1-cell stage before the first cleavage or into two dorsal cells at the 4-cell stage, and the results were compared with non-injected or with CtrlMO-injected siblings. 1 or 2 ng of Cer-S mRNA in total were delivered per embryo, by injecting the vegetal region of all blastomeres at the 4-cell stage, as previously described [37]. The injections are detailed in the figure legends. Some injections included 10 or 20 ng of Dextran Oregon Green 488, MW 10,000, anionic lysine fixable, as tracer (DOG, Molecular Probes, Invitrogen). During injections, embryos were maintained in 4% Ficoll PM400, 1X MBS [43]. Then, they were cultured in 2% Ficoll, 0.1X MBS until sibling controls reached the desired stage. For ectodermal/endomesodermal recombinants, embryos were transferred at stage 9 to agarose plates containing 2% Ficoll, 1X MBS. After removing the vitelline membrane, animal caps were excised with a pair of fine forceps and transferred to recipient embryos in which the animal cap had been removed. After healing for 30 min, embryos were transferred again to 2% Ficoll, 0.1X MBS until sibling controls reached the desired stage. All these solutions contained 50 µg/ml of gentamicin. Embryos were fixed in MEMFA [44] at the indicated stages of development, and were photographed in PBS or were cleared and photographed in Murray's solution (1 volume benzyl alcohol, 2 volumes benzyl benzoate).

We used the Image-Pro Plus software for morphometric measurements and the GraphPad Prism 4 software for t-test analysis.

### Whole-mount in situ hybridization (WMISH) and immunohistochemistry

The preparation of digoxigenin-labelled antisense RNA probes and the WMISH procedure were performed as described previously [45], except that the proteinase K step was omitted. For sectioning, embryos were embedded in Paraplast (Monoject Scientific), and 15–20 µm sagittal and transversal sections were cut on a rotary microtome. They were mounted onto gelatine coated slides. All sections were stained with Hoechst 33258 (Sigma).

Proliferating cells were detected with anti-phosphohistone H3 (Ser10) antibody (Upstate) as follows. After fixation, embryos were washed twice for 15 minutes each with TBSE (10 mM Tris-HCl, 150 mM NaCl, 1 mM EDTA, pH 6.5) and once for 15 minutes with TBSET (TBSE +0.1% Triton X-100). Following an incubation for 3 hours in blocking buffer (TBSET +0.6 % bovine serum albumin), embryos were incubated overnight at 4°C with the primary antibody diluted 1/100 in blocking buffer. After four washes in TBSE of 1 hour each, embryos were incubated for 1 hour in blocking buffer and then, overnight at 4°C with anti-rabbit IgG-HRP (Santa Cruz Biotech), diluted 1/1000 in blocking buffer. After washing twice, for 5 minutes each, with TBS (10 mM Tris-HCl, 150 mM NaCl, pH 6.5), embryos were equilibrated for 30 minutes at room temperature with DAB solution (0.5 mg/ml of 3,3′-diaminobenzidine in 10 mM Tris-HCl, pH 6.5). Staining was revealed with 0.009% of H_2_O_2_ in DAB solution. The reaction was stopped in methanol.

The 3′-fluorescein tag of the morpholinos and the DOG tracer were revealed by immunofluorescence, as previously described [46] [47].

### Whole-mount TUNEL

Whole-mount TUNEL staining was based on a previous protocol [48], with some modifications. After removing the vitelline membrane, embryos were fixed 1 hr in MEMFA, extensively washed in PBS, and stored in ethanol at −20°C. After rehydration in PBS, they were permeabilised in 0,25% Triton X-100/PBS, extensively washed in deionised water, incubated for 1 hr in terminal deoxynucleotidyl transferase (TdT) buffer, and transferred to TdT buffer containing 300 U/ml TdT (Invitrogen) and 1 mM digoxigenin-dUTP (Roche). Incubation was carried out overnight at 23°C. The reaction was stopped in 1 mM EDTA/PBS for 2 hrs at 65°C. Embryos were washed 4 times, 30 minutes each in PBS, and twice in 100 mM maleic acid, 150 mM NaCl, pH 7.5, 5 minutes each, at room temperature. Incubation in blocking reagent and staining with NBT/BCIP were performed as for WMISH [45].

## Results

### Morphogenetic movements, axial elongation, and axial mesodermal regionalization require an intact *foxA4* function

**Figure 1:**
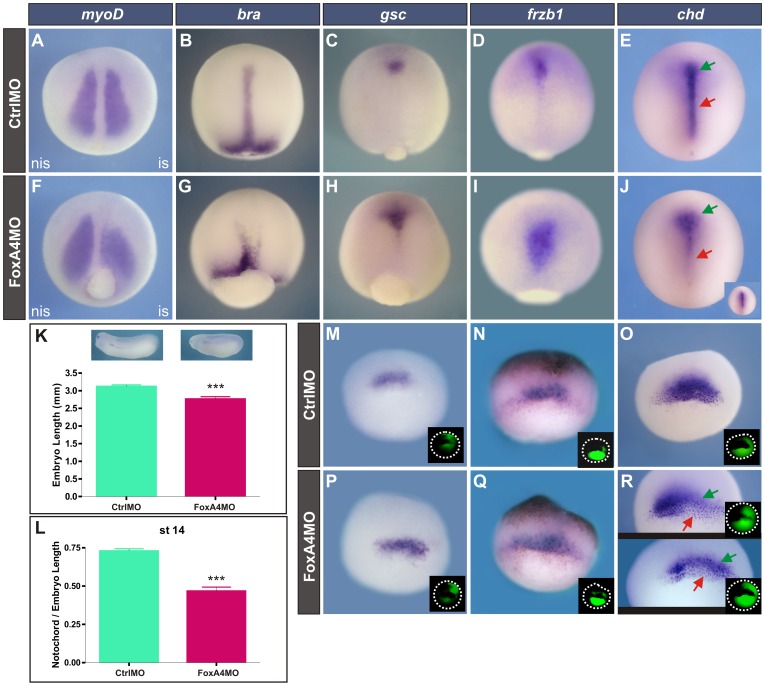
FoxA4 depletion altered the expression of mesodermal markers, impaired axis elongation, expanded the prechordal mesoderm and reduced the notochord. (**A–J**) Expression of mesodermal markers at stage 13, dorsal views. (**A, F**) *myod*. (B, G) *bra*. (C, H) *gsc*. (D, I) *frzb1*. (E, J) *chd*. Embryos were injected into one dorsal cell at the 4-cell stage with 40 ng of CtrlMO (A) or of FoxA4MO (B), or with 20 ng of each MO into both dorsal cells at the 4-cell stage, (CtrlMO: B,C,E; FoxA4MO: G, H, J) or at the 1-cell stage (CtrlMO: D; FoxA4MO: I). The *myoD* domain was shortened on the injected side (is) by FoxA4MO (58%, n = 12) (F). The *bra* domain was split by FoxA4MO (G) (79%, n = 24). The *gsc* domain was posteriorly expanded by FoxA4MO (100%, n = 15) (H). The *frzb1* domain was posteriorly expanded by FoxA4MO (100%, n = 14) (I). The *chd* domain was affected in 100% of the injected embryos (n = 14) (J). Among these, 71% showed an expansion of the anterior *chd* domain, corresponding to the PM (green arrow), while the posterior domain (corresponding to the notochord) was severely reduced (red arrow). In the remaining 29%, the entire axial mesoderm appeared much thinner and shorter than in CtrlMO-injected siblings (inset in J). Similar results were obtained after injection of 20 ng of FoxA4MO into 1-cell stage embryos (Fig. S1B). (**K**) The body length between the cement gland and the tip of the tailbud was measured at tailbud stage in embryos injected before the first cleavage with 20 ng of CtrlMO (green bar, n = 25) or of FoxA4MO (red bar, n = 27). *P*<0.0001, two-tailed t-test. (**L**) Notochord/embryo length ratio measured in the group of embryos corresponding to those shown in (B, G). Green bar, CtrlMO (n = 18); red bar, FoxA4MO (n = 24). *P*<0.0001, two-tailed t-test. (**M–R**) Expression of axial mesodermal markers at stage 10.25. (M, P) *gsc*. (N, Q) *frzb1*. (O, R) *chd*. Embryos were injected with 20 ng of CtrlMO (M–O) or of FoxA4MO (P–R). Injections were performed unilaterally in 1 cell at the 2 or 4-cell stage or at the 1-cell stage, giving similar results. Unilateral injections are shown here. The injected side was identified by DOG fluorescence and is oriented to the right (insets in M–R). In gastrulae, we were able to observe an expansion of *gsc* (58%, n = 40; P) and of *frzb1* domains (88%, n = 33; Q). *chd* expression was reduced in the less involuted, superficial cells on the injected side (red arrows), but a cloud of deep cells expressing *chd* persisted (green arrows), and even appeared to be expanded on the injected side in some embryos, like the one shown below (50%, n = 10) (R).

To study the effect of knocking-down *foxA4* on axial development, embryos were injected with FoxA4MO [36] or CtrlMO. Their morphology and the expression of molecular markers were analysed at stage 13. Embryos injected with FoxA4MO failed to close the blastopore (Fig. 1). At neural plate stage, *myoD* is normally expressed in the paraxial mesoderm in two domains adjacent to the developing notochord (Fig. 1A) [49]. In unilaterally injected embryos, the *myoD* domain was wider and shortened on the injected side after knock-down of *foxA4* (Fig. 1F).

Different strategies that impair convergent-extension movements consistently result in shortened A–P axis, since these morphogenetic movements normally drive the elongation of the body [50] [51] [52] [53] [54]. Measurement of the body length confirmed that the A–P axis was significantly shortened in FoxA4-depleted larvae (Fig. 1K). Therefore, we compared the expression of genes that oppositely control two distinct morphogenetic movements in contiguous regions of the axial mesoderm. The T-box transcription factor *brachyury* (*bra*) is expressed in the chordamesoderm (Fig. 1B), where it is required for convergent extension and to inhibit the active cell migration behaviour typical of the PM [55]. The transcriptional repressor *goosecoid* (*gsc*) is expressed in the PM (Fig. 1C), where it is required to repress *bra*, promoting active cell migration [56] [57] [58].

To study if knock-down of *foxA4* affects the expression of these axial markers at neural plate stage, we delivered FoxA4MO more homogeneously throughout the embryo. In FoxA4 morphants which were bilaterally injected, the *bra* domain was split in two branches and the notochord was significantly shorter at the early neural plate stage (Fig. 1G, L) in comparison to CtrlMO-injected siblings (Fig. 1B, L). Complementarily, *gsc* expression was expanded in a triangular form, extending more posteriorly (Fig. 1H) than in CtrlMO-injected embryos (Fig. 1C). These results suggest that FoxA4 depletion might be favouring the PM fate at the expense of posterior axial mesoderm. To corroborate these observations, we analyzed the expression of *chordin* (*chd*) and *frzb1* in embryos with a homogenous delivery of the morpholino. At the early neural plate stage, when prechordal mesoderm and notochord have normally definitively segregated, it can clearly be seen that *chd* is expressed along the entire axial mesoderm, both in the notochord (red arrow, Fig. 1E) and in the PM (green arrow, Fig. 1E), whereas *frzb1* overlaps *chd* in the PM but it is not expressed in the notochord (Fig. 1D) [59] [60] [61]. In FoxA4 morphants the *frzb1* domain was remarkably expanded, and similarly to *gsc*, it was extended caudally (Fig. 1I), invading territories which are normally occupied by the notochord. Notably, this was correlated with an expansion of the anterior *chd* domain (corresponding to the PM; Fig. 1J, green arrow), while the posterior domain (corresponding to the notochord) was severely reduced in comparison to Ctrl MO-injected siblings (Fig. 1J, red arrow) (71%, n = 14). A smaller proportion of FoxA4 morphants showed the entire axial mesoderm affected, which appeared much thinner and shorter than in Ctrl MO-injected siblings (29%, n = 14) (Fig. 1J, inset).

At early gastrula stages, injection of FoxA4MO also expanded the *gsc* and *frzb1* domains (Fig. 1P, Q) and reduced the expression of *chd* (Fig. 1R). This reduction in *chd* occurred in the more superficial, less involuted cells, corresponding to the presumptive notochord (Fig. 1R, red arrows). However, we observed a deep cloud of *chd +* refractory cells on the injected side, corresponding to the deep cells of the organiser, which are known to give rise to the PM (Fig. 1R, green arrows). These results indicate that in FoxA4 morphants, the precursors of the axial mesoderm are already affected in the organiser.

**Figure 2:**
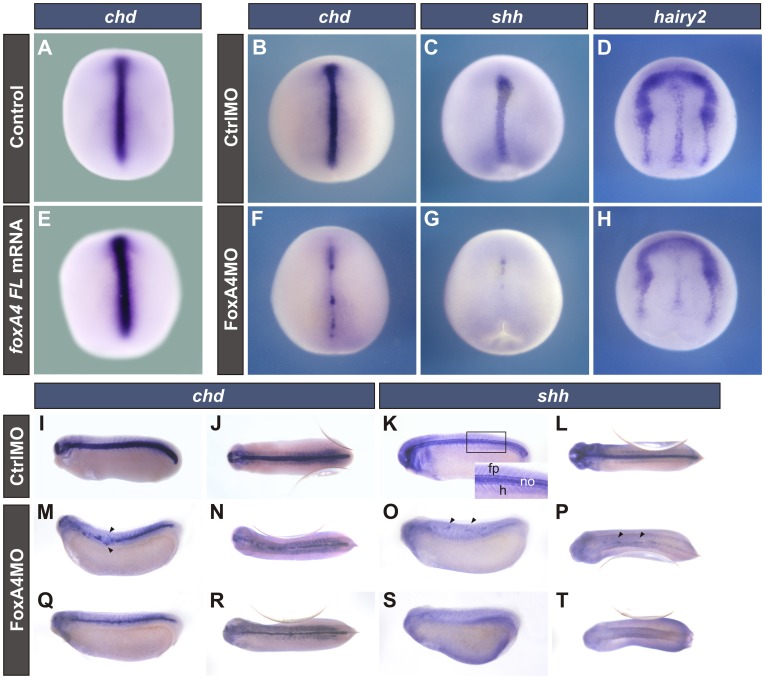
FoxA4MO disrupted DML formation. (**A–H**) Stage 15 embryos left uninjected (A) or injected with 0.5 ng of *foxA4FL* mRNA (E), 40 ng of CtrlMO (B–D) or of FoxA4MO (F–H) before the first cleavage. (**I–T**) Tailbud stage embryos injected into two dorsal cells at the 4-cell stage with 20 ng of CtrlMO or of FoxA4MO. The descendants of these cells give rise to the DML and the cephalic region. Expression of *chd* (A, B, E, F, I, J, M, N, Q, R), *shh* (C, G, K, L, O, P, S, T), *hairy2* (D, H). The inset in K shows a magnified view of the area depicted by the rectangle; fp, floor plate; no, notochord; h, hypochord. Embyos injected with *foxA4FL* mRNA showed a thicker notochord than control siblings, as revealed by *chd* expression (87%, n = 15) (E). The percentage of embryos injected with FoxA4MO showing the indicated changes in the corresponding markers is indicated between parentheses, as follows. At neural plate stage, the expression of *chd* (90%, n = 20) (F) and *shh* (54%, n = 22) (G) was patchy, and *hairy2* expression was reduced in the FP domain (60%, n = 23) (H). At stage 26, the *chd* domain was patchy and/or thinner (80%, n = 21) (M, N, Q, R). Arrowheads point to *chd* + cells in dorsal and ventral positions with respect to a notochordal gap. *Shh* was also patchy (arrowheads, O, P) or completely abolished (S,T) (86%, n = 22). In overall, tailbuds injected with FoxA4MO showed reduced heads (89%, n = 51) (M, Q, O, S). (A–H, J, L, N, P, R, T) dorsal views; (I, K, M, O, Q, S) lateral views.

In contrast to what happened in FoxA4 morphants, after overexpression of *foxA4FL* mRNA, *chd* expression in neurulae revealed that the notochord was consistently thicker (Fig. 2E). A similar effect was obtained after injection of *foxA4CDS* mRNA (Fig. S1C, K). This mRNA, which lacks the target sequence for FoxA4MO, was able to rescue the effects of FoxA4MO on *chd* expression. While most of the embryos injected with 20 ng of FoxA4MO showed the typical expansion of the anterior/prechordal domain and the suppression of the posterior/notochordal domain at early neural plate stage (83%, Fig. S1B, K), none of the embryos co-injected with 20 ng of FoxA4MO +0.5 ng of *foxA4CDS* mRNA showed this phenotype. Instead, they were similar to uninjected controls (total rescue, 50%, Fig. S1D, K) or showed a DML slightly thinner but of similar length than that of the uninjected controls (partial rescue, 50%; Fig. S1E, K). These results indicate that the effects of FoxA4MO on DML development are specific.

In conclusion, our results indicate that *foxA4* is necessary for axial elongation and normal morphogenetic movements. Remarkably, it is also involved in the segregation between anterior and posterior axial mesodermal precursors, restricting the development of PM in favour of the notochord.

### Depletion of FoxA4 causes disruption of the DML structures

Among the structures that compose the trunk DML, the notochord expresses *chd* and *sonic hedgehog* (*shh*). The FP initially expresses *shh* and *hairy2*, whereas *chd* transcripts are also found at tailbud stages. The hypochord expresses *shh* at tailbud stages (Fig. 2, S2) [62] [63] [64] [65]. To elucidate whether the notochord disruption observed in FoxA4 morphants was due to a general disturbance in the development of the DML, we analysed the expression of these markers. At more advanced neurula stages than those analysed in Fig. 1, the expression of *chd* and *shh* was reduced and patchy in FoxA4 depleted embryos (Fig. 2F, G) in relation to CtrlMO-injected siblings (Fig. 2B, C). *Hairy2* expression was also reduced in the FP domain (Fig. 2H) in comparison to embryos injected with CtrlMO (Fig. 2D) but was unaffected at the neural plate border, from where neural crest cells arise [66]. Therefore, FoxA4 depletion disrupted the organisation of the DML.

The patchy aspect of the DML markers persisted in FoxA4 morphant tailbuds, which showed reduced heads (compare Fig. 2M, Q, O, S with Fig. 2I, K). The distribution of *chd* transcripts revealed that the notochord was frequently interrupted and/or notably thinner than in CtrlMO-injected siblings (compare Fig. 2M, N, Q, R with Fig. 2I, J). Groups of *chd* expressing cells were often found in dorsal and ventral positions with respect to the notochordal gaps (arrowheads, Fig. 2M). Transverse sections of these embryos showed that these cells were located within the ventral neural tube or the dorsal midline of the endoderm, possibly occupying the place of the FP and the hypochord, while at the same level, the notochord was disorganised or absent (Fig. S2). *Shh* expression was severely reduced to small patches of cells (arrowheads, Fig. 2O, P) or absent from the DML (Fig. 2S, T).

All these results indicate that the three components of the trunk DML were affected from early stages of specification. In conclusion, *foxA4* is a major factor necessary for the development of the *Xenopus* DML. However, the expression of axial markers was not completely abolished in all the embryos injected with FoxA4MO.

### Depletion of FoxA4 changes the fate of dorsal mesodermal and neuroectodermal cells

**Figure 3:**
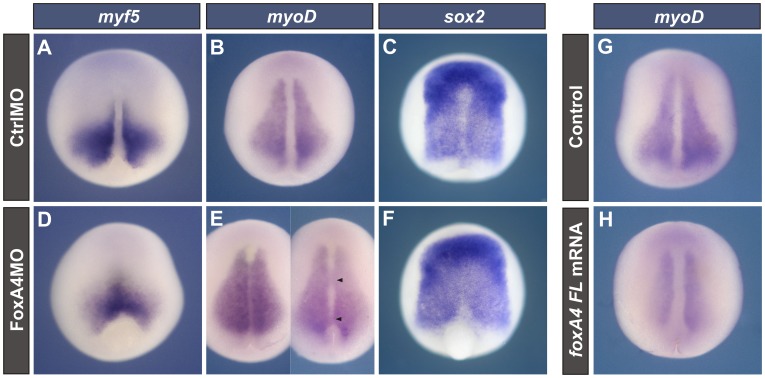
The DML is invaded by paraxial mesoderm and neuroectoderm in FoxA4 morphants. Dorsal views of embryos at neural plate stage hybridised with *myf5* (A, D), *myoD* (B, E, G, H), or *sox2* probes (C, F). They were injected before the first cleavage with 20 ng CtrlMO (A–C) or of FoxA4MO (D–F), 0.25 ng of *foxA4FL* mRNA (H), or left uninjected (G). In FoxA4 morphants, *myf5* (D) and *myoD* expression (E) was found in the DML (40%, n = 23; 56%, n = 23, respectively). The left embryo in E shows that *myoD* expression invaded the DML along the A–P axis. The right embryo in E shows stretches of the *myoD* domains fused at the midline (arrowheads). *Sox2* transcripts obliterated the prospective FP (79%, n = 19) (F). In embryos injected with *foxA4FL* mRNA, *myoD* expression was reduced, and the medial borders of the bilateral domains were more separated (H, 42%, n = 26) than in control siblings (G).

Since FoxA4 morphants revealed severe gaps in the DML, we wondered whether the FP and the axial mesoderm were being replaced by surrounding tissues. Therefore, we analysed the expression of paraxial mesoderm and neural plate markers, which are not normally expressed in the DML. In neurulae, *myf5* and *myoD* are expressed in the presomitic mesoderm, but *myf5* transcripts are only found in the posterior region, while *myoD* expression extends along the trunk (Fig. 3A, B) [49] [67]. The transcription factor *sox2* is a marker of neuroectoderm specification (Fig. 3C) [68] [69]. In FoxA4 depleted neurulae, the bilateral domains of *myf5* fused in a single domain, while the intervening midline completely disappeared (Fig. 3D) or was invaded by *myoD* + cells, while the bilateral *myoD* domains, although still discernible, were closer to each other or were fused in stretches at different levels of the trunk (Fig. 3E). Similar to what happened with the paraxial mesoderm markers, *sox2* expression almost obliterated the midline of the neural ectoderm (Fig. 3F), while in CtrlMO-injected siblings, *sox2* transcripts were excluded from the prospective FP (Fig. 3C).

In contrast to the observations in the FoxA4 morphants, after injection of *FoxA4FL* mRNA, the *myoD* bilateral domains were reduced and were more separated than in control embryos (compare Fig. 3H with 3G). A similar effect was obtained after injection of *foxA4CDS* mRNA (Fig. S1H). This mRNA was able to rescue the effects of FoxA4MO on the pattern of *myoD* expression. While the majority of the embryos injected with 20 ng of FoxA4MO showed the typical fusion between the bilateral *myoD* domains (73%, Fig. S1G), the proportion of embryos showing this phenotype notably decreased after co-injection of 20 ng of FoxA4MO +0.5 ng of *foxA4CDS* mRNA (29%, Fig. S1L). Instead, the majority of the co-injected embryos (71%) were either similar to uninjected siblings (32%, Fig. S1I, L) or showed an overexpression phenotype (39%; Fig. S1J, L), indicating that the co-injected *foxA4CDS* mRNA, which lacks the target sequence for FoxA4MO, reversed the effect of FoxA4MO. Together with the rescue experiments analysed with *chd*, these results indicate that the effects of FoxA4MO on DML and paraxial mesoderm markers are specific.

Our results could be attributable to an increase in cell death of DML precursors, with paraxial mesodermal and neuroectodermal cells simply occupying their place. However, we observed no difference in the number of apoptotic cells between FoxA4MO and CtrlMO-injected embryos at early neurula and early tailbud stages, as assessed by the TUNEL assay. In fact, the DML was completely devoid of apoptosis (Fig. S3A–D). Therefore, DML gaps were not produced by an increase in cell death in FoxA4 morphants in the stages analysed.

Alternatively, the disruption of the DML could be attributable to a reduction of proliferation of its precursors. However, we did not observe differences in the number of mitotic nuclei between CtrlMO- and FoxA4MO-injected embryos, as revealed by Phosphohistone H3 immunohistochemistry (Fig. S3E, F, G).

Therefore, our results indicate that FoxA4 depletion promotes a change in DML precursors to contiguous fates, favouring the paraxial mesoderm. Hence, these tissues occupy the axial territory, together with the neural ectoderm, which invades the domain of the missing FP. We conclude that *foxA4* is necessary for restricting the paraxial mesoderm in favour of the DML.

### *FoxA4* inhibition favours anterior fates at neural plate stage

**Figure 4:**
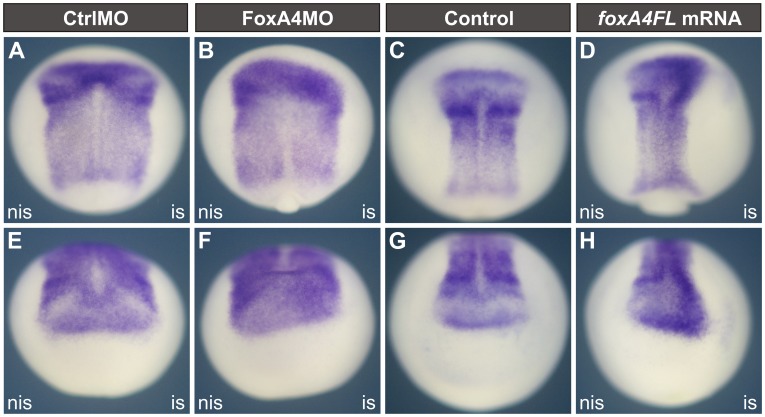
Knock-down and overexpression of *foxA4* shifted the anterior boundary of *sox2* in opposite directions. *Sox2* expression in stage 14–15 embryos injected with 20 ng of CtrlMO (A, E) or FoxA4MO (B, F) or with 0.5 ng of *foxA4FL* mRNA (D, H) into one cell at the 2 cell stage, or left uninjected (C, G). FoxA4MO produced a caudal shift (62,5%, n = 16) (B, F). *FoxA4FL* mRNA produced an anterior shift of the anterior boundary of *sox2* (52%, n = 23) (D, H). (A–D) dorsal views; (E–H) anterior views; is, injected side; nis, non-injected side.

Depletion of FoxA4 produced a caudal shift of the anterior neural plate boundary, as revealed by *sox2* expression (Fig. 4B, F), whereas *foxA4FL* overexpression expanded the neural plate anteriorly (Fig. 4D, H). Therefore, we wondered whether the A–P pattern of the CNS was affected.

The rostral forebrain is an evolutionary acquisition of vertebrates, since anatomical homologues of this anterior compartment are not present in lower chordates. It gives rise to the telencephalon and to rostral diencephalic derivatives, such as the eyes and the hypothalamus, while the caudal forebrain gives rise to caudal diencephalic derivatives [70] [71]. The expression of the transcription factor *Xanf1* in the anterior neural plate ensures the development of the rostral forebrain by repressing the caudal forebrain regulator *otx2* [70] [72].

**Figure 5:**
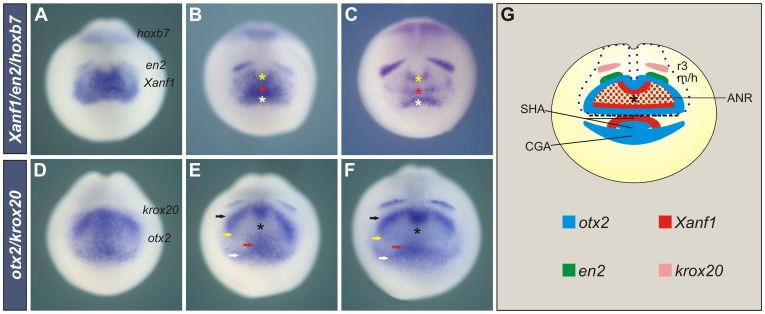
*Xanf1* and *otx2* progressively establish complementary domains. Normal expression of *Xanf1*, *en2* and *hoxb7* (A, B, C) and *otx2* and *krox20* (D–F) in progressively older neural plate stage embryos in anterior views. Yellow asterisks, posterior border of *Xanf1* in the ANR; red asterisks, anterior border of *Xanf1* in the ANR; white asterisks, SHA; black asterisks, expression hole in the neural *otx2* subdomain; black arrows, caudal diencephalic-mesencephalic stripes demarcating the posterior border of the *otx2* neural subdomain; yellow arrows, stripe corresponding to the anterior border of *otx2* in the ANR; red arrows, SHA; white arrows, CGA. (G) Diagram summarising the expression patterns of *otx2*, *Xanf1*, *en2*, and *krox20* in an anterior view of embryos at the stages shown in B, C, E, F. ANR, anterior neural ridge; r3, third rhombomere; m/h, midbrain/hindbrain boundary.

**Figure 6:**
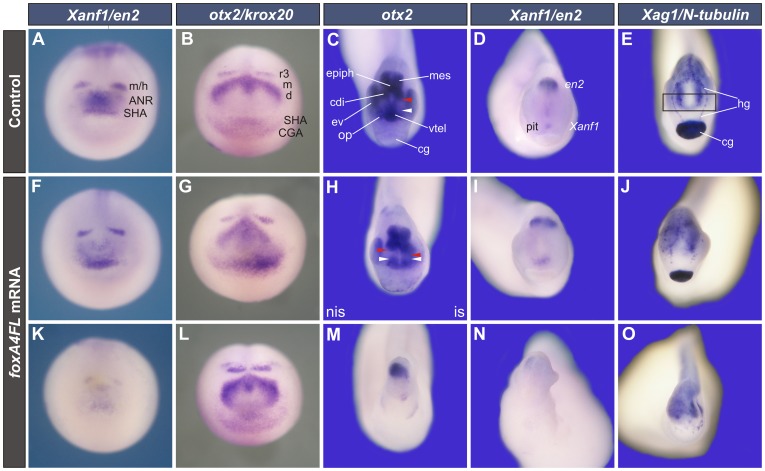
*FoxA4* overexpression produced complementary effects on *Xanf1* and *otx2* and disfavoured anterior development. (**A, B, F, G, K, L**) Anterior views of early neural plate stage embryos analysed by WMISH with the following markers: *Xanf1* and *en2* (A, F, K), *otx2* and *krox20* (B, G, L). Embryos were injected with 0.25 ng (F, K) or 1 ng (G, L) of *foxA4FL* mRNA before the first cleavage, or were left uninjected (A, B). *FoxA4* overexpression affected *Xanf1* expression in 83% of the injected embryos (n = 23) as follows: it was reduced in the ANR but not in the SHA (48%, n = 23) (F), or it was reduced in the whole domain (35%, n = 23) (K). The expression hole of the *otx2* domain was filled with *otx2* transcripts (42%, n = 19) (G); the *otx2* domain was reduced, but the caudal diencephalic/mesencephalic stripes were expanded (89%, n = 19) (L). (**C–E, H–J, M–O**) Anterior views of tailbuds (stage 27/28) injected with 0.25 ng (H, I, M, N) or 0.5 ng (J, O) of *foxA4FL* mRNA before the first cleavage (I, J, M–O) or into one cell at the 2-cell stage (H), or left uninjected (C, D, E). Expression of *otx2* (C,H,M), *Xanf1* and *en2* (D, I, N), *Xag1*/*N-tubulin* (E, J, O). After overexpression of *foxA4*, the caudal diencephalic *otx2* subdomain was expanded anteriorly (54%, n = 13) (H) or the telencephalic *otx2* subdomain disappeared (40% n = 10) (M). The pituitary *Xanf1* domain was expanded (33%, n = 15) (I). *N-tubulin* expression in the rostral forebrain (rectangle in E) was deleted and the *Xag1* + cement and hatching glands were reduced (J) or the cement gland was absent (O) (54%, n = 24). The most severe phenotypes presented head truncations (23%, n = 13) (M,N,O). Red arrowhead, anterior limit of the caudal diencephalic *otx2* subdomain; white arrowhead, posterior limit of the ventral telencephalic *otx2* subdomain; vtel, ventral telencephalic *otx2*; cdi, caudal diencephalic *otx2*; mes, mesencephalic *otx2*; op, olfactory placode; epiph, epiphisis; ev, eye vesicle; cg, cement gland; hg, hatching gland cells; pit, pituitary anlage; is, injected side; nis, non-injected side.

We observed that the complementary patterns of *Xanf1* and *otx2* in the anterior neural plate that were previously described [70] [72] are extensive to the stomodeal-adenohypophyseal-cement gland anlage (SHCGA) in the ectoderm (Fig. 5). Initially, the SHCGA partially overlaps with the anterior neural ridge (ANR) during the gastrula to neurula transition, and later they gradually segregate [73]. At neural plate stage, the domain of *Xanf1* has a horseshoe shape (Fig. 5A) [70] [74] [75]. Around stage 15, transcripts of *Xanf1* progressively accumulate in three stripes (Fig. 5B, C, G). We presume that the most anterior one (white asterisk) corresponds to the stomodeal-adenohypophyseal anlage (SHA), which is beginning to segregate from the ANR and from the cement gland anlage (CGA). Indeed, at later stages, *Xanf1* transcripts only persist in the prospective anterior pituitary (Fig. 6D) [75]. The other two *Xanf1* stripes correspond to the anterior border (red asterisk, Fig. 5B, C) and to the posterior border (yellow asterisk, Fig. 5B, C) of the ANR (Fig. 5G). At this stage, *Xanf1* marks the future ventral and dorso-rostral part of the diencephalon and the prospective telencephalon [70] [74] [75].

Initially, *otx2* is expressed in a relatively uniform region comprising the anterior neural plate and the adjacent ectoderm, including the SHCGA (Fig. 5D), which progressively segregate (Fig. 5E, F). The neural aspect of *otx2* is then resolved into a subdomain that encircles an expression hole (black asterisk, Fig. 5E, F, G), which coincides with the ANR *Xanf1* subdomain (Fig. 5B, C, G). The *otx2* neural subdomain is limited anteriorly by a transverse stripe demarcating the ANR, corresponding to telencephalic precursors (yellow arrow, Fig. 5E, F), and posteriorly, by a conspicuous stripe in the presumptive mesencephalic and caudal diencephalic region (black arrows, Fig. 5E, F) [70] [72] [76] [77]. The ectodermal component is resolved into the SHA (red arrow, Fig. 5E, F) and the CGA (white arrow, Fig. 5E, F). In conclusion, the complementary patterns of *Xanf1* and *otx2* encompass the whole anterior-most region of the embryo (Fig. 5G).

Immediately juxtaposed to the posterior border of *otx2*, *engrailed-2* (*en2*) is expressed in two bilateral stripes demarcating the future midbrain/hindbrain boundary [78] (Fig. 5A–C, G). *Krox20* is expressed in the hindbrain, initially marking the third rhombomere (Fig. 5D–G) and later, it also appears in the fifth rhombomere [79]. *Hoxb7* is expressed in the spinal cord, with an anterior limit in the caudal hindbrain (Fig. 5A–C) [80] [81].

**Figure 7:**
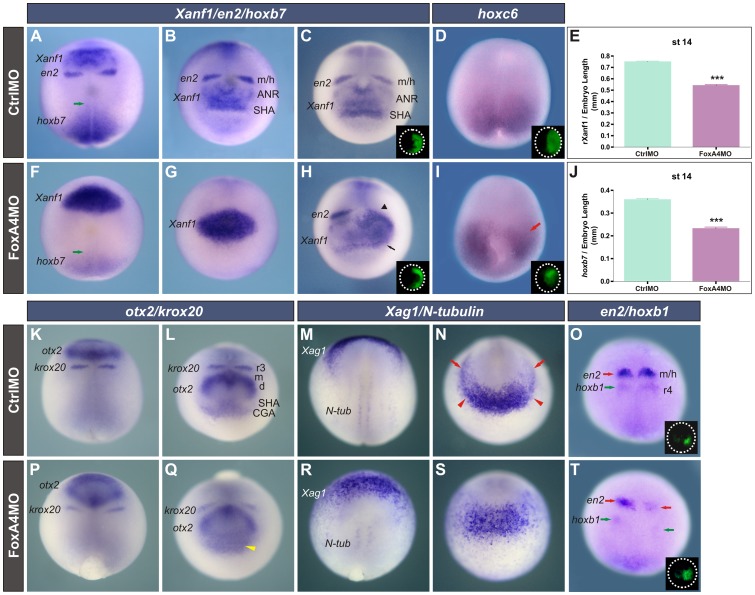
*FoxA4* inhibition affected anterior ectodermal/neural specification. Embryos at early neural plate stage injected with 20 ng of CtrlMO or of FoxA4MO before the first cleavage (A, B, F, G, K–N, P–S) or unilaterally injected into 1 cell at the 2-cell stage (C, D, H, I, O, T). The injected side was determined by DOG fluorescence (insets). Embryos were hybridised with the following probes: *Xanf1*, *en2*, and *hoxb7* (A–C, F–H); *hoxc6* (D, I); *otx2* and *krox20* (K, L, P, Q); *Xag1* and *N-tubulin* (M, N, R, S); *en2* and *hoxb1* (O, T). Green arrows in (A, F) point to the anterior limit of *hoxb7*. At neural plate stage, *Xag1* marks the cement gland anlage (indicated between red arrowheads in N), and the hatching gland primordia (red arrows in N) [98] [90]. Black arrowhead in (H), caudal shift of *Xanf1* and fusion with the *en2* stripe. Black arrow in (H), fusion of the SHA and the ANR. Red arrow in (I), down-regulation of *hoxc6* on the injected side. Yellow arrowhead in (Q), diffuse CGA and anterior border of the ANR. Notice the down-regulation and the caudal shift of *en2* (red arrows) and *hoxb1* (green arrows) by comparing the injected side (right) with the non-injected side (left) in the FoxA4 morphant in (T) and with the CtrlMO-injected embryo in (O). (E) Ratio between the distance from the posterior limit of *Xanf1* to the blastopore and the total length of the embryo in the groups shown in A, B, F, G (r-Xanf1). The ratio was significantly lower in FoxA4 morphants; *P*<0.0001, two-tailed t-test. (J) The ratio between the length of the *hoxb7* domain and the embryo’s length was significantly lower in FoxA4 morphants, as measured in the groups shown in A, B, F, G; *P*<0.0001, two-tailed t-test. ANR, anterior neural ridge; CGA, cement gland anlage; SHA, stomodeal-adenohypophyseal anlage; m/h, midbrain/hindbrain boundary; m, d, presumptive mesencephalic and caudal diencephalic regions expressing *otx2*; r3, third rhombomere; r4, fourth rhombomere. (A, D, F, I, K, M, O, P, Q, R, T) are dorsal views; (B, C, G, H, L, N, Q, S) are anterior views.

**Figure 8:**
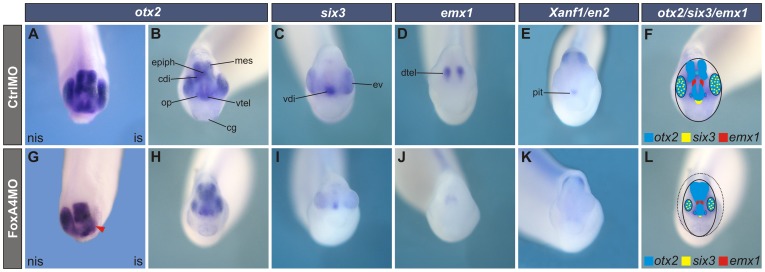
Depletion of FoxA4 led to head defects at tailbud stages. Anterior views of stage 27/28 embryos injected with 20 ng of CtrlMO (A–E) or FoxA4MO (G–K). Embryos were injected into one dorsal cell at 4-cell stage (A, G) or prior to the first cleavage (B–E, H–K). Expression of *otx2* (A, B, G, H), *six3* (C, I), *emx1* (D, J) and *Xanf1* (E, K). Results are summarised in F, L. In embryos unilaterally injected with FoxA4MO, the telencephalic and caudal diencephalic *otx2* subdomains were fused in the injected side (red arrowhead), and the eye was lost (70%, n = 24) (G). When FoxA4MO was homogenously distributed, the *otx2* domain was reduced, the telencephalic subdomain was fused in the midline and also, with the caudal diencephalic subdomain (80%, n = 20) (H). FoxA4MO reduced the expression domains of *six3* (87%, n = 23) (I), *emx1* (80%, n = 26) (J) and *Xanf1* (70%, n = 20) (K). dtel, dorsal telencephalon; vtel, ventral telencephalic *otx2*; cdi, caudal diencephalic *otx2*; mes, mesencephalic *otx2*; op, olfactory placode; epiph, epiphisis; ev, eye vesicle; cg, cement gland; pit, pituitary anlage; is, injected side; nis, non-injected side. The black line in F, L represents the contour of the head. For comparison, the head contour of the CtrlMO-injected embryo was projected on the FoxA4 morphant diagram (dotted black line in L).

**Figure 9:**
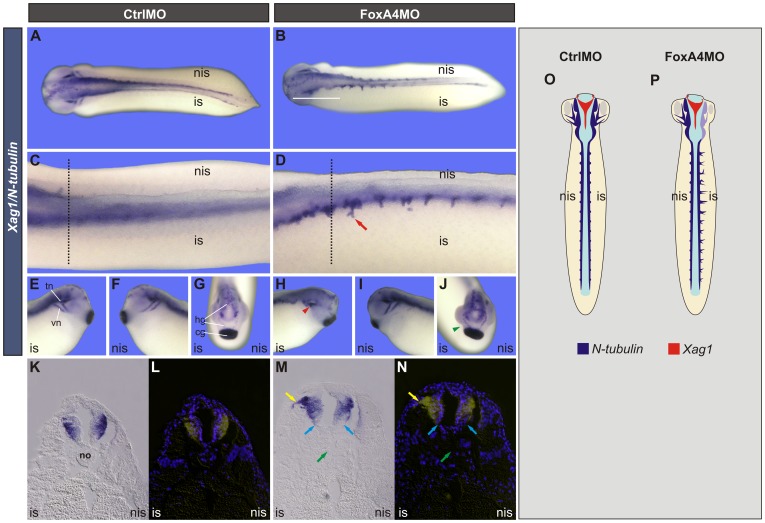
FoxA4 depletion altered neurogenesis along the A–P axis. (**A–J**) *N-tubulin* and *Xag1* expression in stage 27/28 embryos injected with 40 ng of CtrlMO (A, C, E–G) or FoxA4MO (B, D, H–J) into one cell at the 2-cell stage. (A, B) Dorsal views. (C, D) Magnified images of the trunk region of another pair of CtrlMO (C) and FoxA4MO (D) injected embryos, shown in dorso-lateral views to facilitate comparisons. The red arrow points to an *N-tubulin* + projection on the injected side. (E, F, H, I) Lateral views of the head region. (G, J) Anterior views. *N-tubulin* is expressed in the trigeminal (tn) and vestibulocochlear (vn) nerves. In FoxA4MO-injected embryos, the tn and vn were disturbed (red arrowhead) (H), as revealed by *N-tubulin*; the cement gland (cg) was bent and closer to the forebrain, and the hatching gland (hg) was shortened on the FoxA4-depleted side, as revealed by *Xag1* (green arrowhead) (J) (90%, n = 21). (**K–N**) Transverse sections at the levels indicated by dotted black lines in C,D, shown in bright field (K, M) and their corresponding nuclear Hoescht fluorescence (L, N). Bright field images were processed with Adobe Photoshop CS2 in order to superimpose the *N-tubulin* expression (yellow) to the Hoescht fluorescence. Yellow arrows point to the projection emerging from the dorsal neural tube; cyan arrows point to both sides of the ventral neural tube; green arrows point to the place left by the disorganised or absent notochord; no, notochord; is, injected side; nis, non-injected side. (O, P) Summary of the results shown in A–J.

When *foxA4* was homogenously inhibited, we observed complementary changes in the expression of *Xanf1* and *otx2*. *Xanf1* was strongly up-regulated in such way that its subdomains were no longer evident. Its caudal border shifted posteriorly, fusing to the *en2* stripes (100%, n = 22) (compare Fig. 7F, G with Fig. 7A, B). The relative distance between the posterior limit of *Xanf1* and the caudal end of the embryo was significantly shortened (Fig. 7E), confirming the caudal shift. Similar results were obtained in embryos unilaterally depleted from FoxA4 (arrowhead, Fig. 7H). Since in these embryos the *Xanf1* subdomains could be recognised on the non-injected side, we noticed that on the injected side, the SHA stripe was also caudally shifted and almost fused with the ANR domain (71,5% n = 21) (arrow, Fig. 7H). Complementarily to the increase in *Xanf1*, the diencephalic-mesencephalic *otx2* stripes were thinner than in CtrlMO-injected siblings (compare Fig. 7P, Q with Fig. 7K, L). The CGA and ANR subdomains were more diffuse and fused, while the SHA was not longer distinguished (93%, n = 29) (yellow arrowhead, Fig. 7Q). This was confirmed with the cement gland marker *Xag1* [82], whose transcripts were more scattered and showed a caudal expansion of the domain (86%, n = 21) in relation to CtrlMO-injected siblings (compare Fig. 7R, S with Fig. 7M, N), thus complementing the caudal retraction of the anterior neural plate border (Fig. 4B, F). The disappearance of the SHA is consistent with the ulterior absence of the pituitary *Xanf1* expression in tailbuds (Fig. 8K) and with the posteriorwards bending of the cement gland, which was closer to the forebrain in the FoxA4 depleted side (green arrowhead, Fig.9J).

**Figure 10:**
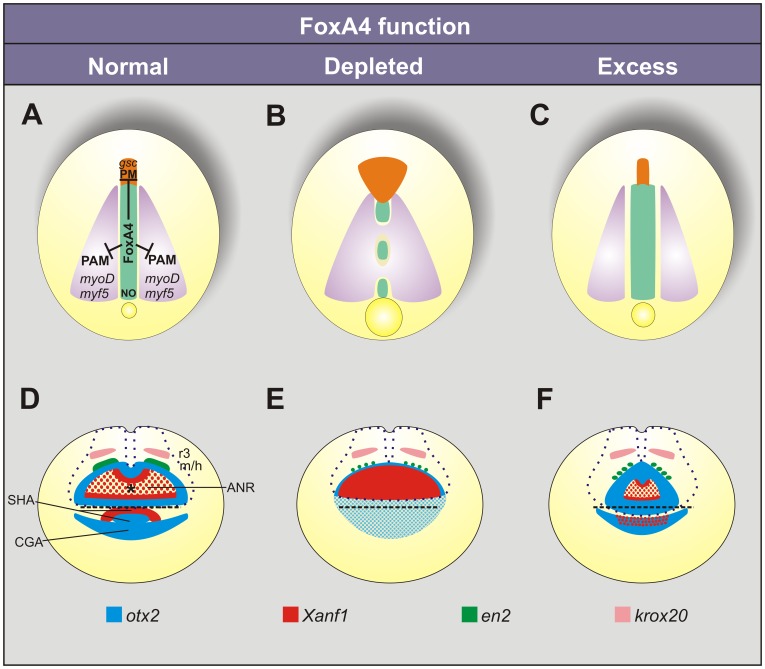
Summary of the phenotypes after manipulating *foxA4* function in *Xenopus* embryos. The diagrams show the phenotypes in embryos with normal (A, D), depleted (B, E), or excess levels (C, F) of *foxA4* function, as revealed by the expression patterns of *bra, chd, myf5/myoD*, *gsc*, *frzb1, otx2*, *Xanf1*, *krox20* and *en2* at neural plate stage. (A–C) In DML development, *foxA4* is necessary to restrict the paraxial mesodermal (PAM) and the prechordal mesodermal (PM) fates, allowing notochord (NO) development. (D–F) Controlled levels of *foxA4* function are necessary for the correct specification and segregation of the anterior neural and ectodermal anlagen. ANR, anterior neural ridge; SHA, stomodeal-adenohypophyseal anlage; CGA, cement gland anlage; r3, third rhombomere; m/h, midbrain/hindbrain boundary. The asterisk in D marks the expression hole left by *otx2* in the ANR, which is occupied by *Xanf1*. The dotted blue line (D–F) represents the contour of the neural plate. The dashed black line (D–F) represents the anterior boundary of the neural ectoderm. (A–C) Dorsal views. (D–F) Anterior views. See text for details.

We conclude that the *Xanf1* subdomain corresponding to the ANR was expanded at the expense of the caudal diencephalic-mesencephalic *otx2* subdomain, indicating an expansion of the presumptive anterior forebrain compartment at the expense of the presumptive caudal forebrain and midbrain. The anterior ectoderm/neural ectoderm boundary became diffuse, with a posterior shift of the CGA at the expense of the SHA and of the anterior border of the neural plate. These results are summarised in Fig. 10E. Therefore, the attenuation of *foxA4* produced a general anteriorisation involving both the ectoderm and the neural ectoderm, with complementary changes in the expression of *otx2* and *Xanf1*.

In addition, we also observed alterations in posterior markers when we attenuated *foxA4* function. *N-tubulin* is a marker of differentiated neurons [83], and the stripes of *N-tubulin* + primary motorneurons in the trunk were shorter in FoxA4 morphants (Fig. 7R) than in CtrlMO-injected siblings (Fig. 7M), while the expression of *hoxb7* was retracted caudally (Fig. 7F, J) in comparison to CtrlMO-injected embryos (Fig. 7A, J), as well as that of *hoxc6*, which marks the presumptive spinal cord [84] (90%, n = 10; Fig. 7I, red arrow). Moreover, the hindbrain was also affected, since *hoxb1*, which is normally expressed in the presumptive 4^th^ rhombomere [80] (Fig. 7O), was down-regulated and shifted caudally (100%, n = 9; Fig. 7T) and the expression of *krox20* in r5 was inhibited (Fig. S4B, H). In overall, our results indicate that depletion of FoxA4 promotes anterior neural specification while disfavours posterior neural development. In conclusion, *foxA4* is required for the establishment of the normal A–P patterning of the neural plate.

### *FoxA4* overexpression inhibits anterior development and rescues the anteriorisation produced by FoxA4MO

At neural plate stage, overexpression of *foxA4FL* caused quite the opposite effects in comparison to FoxA4 depletion. *Xanf1* was drastically down-regulated in the most severely affected embryos, while in milder phenotypes, the SHA *Xanf1* domain persisted and was sometimes enlarged, but the expression in the ANR was still inhibited (compare Fig. 6F, K with 6A). While the ANR *Xanf1* domain tended to collapse, *otx2* was up-regulated and its expression hole tended to disappear (compare Fig. 6G, L with 6B). These results are summarised in Fig. 10F.

In tailbuds, *otx2* normally marks the presumptive ventral telencephalon, mesencephalon and caudal diencephalon, leaving a gap of lower expression between the telencephalon and caudal diencephalon, corresponding to the rostral diencephalon (area between white and red arrowheads, Fig. 6C and non-injected side in Fig. 6H) [70] [76] [85]. Unilaterally *foxA4FL*-injected embryos revealed an anterior expansion of the *otx2* caudal diencephalic domain (red arrowhead on the injected-side, Fig. 6H) concomitant with an obliteration of the *otx2* telencephalic-diencephalic gap, indicating that more posterior brain fates were favoured at the expense of the rostral diencephalon. This is consistent with the reduction of the eye on the injected side, since the eye vesicle derives from the rostral diencephalon [70] [71]. When *foxA4FL* mRNA was homogenously delivered, the head was truncated in the most extreme phenotypes (Fig. 6M, N, O). These embryos lacked eyes, and the cement gland and the telencephalic *otx2* subdomain disappeared. Only the posterior *otx2* expression remained, indicating that the rostral forebrain was deleted. This is consistent with the reduction or absence of the eye vesicle, and with the absence of the most anterior expression of *N-tubulin* (compare Fig. 6E, J, O).

In some milder phenotypes, we could observe an expansion of the pituitary or a reduction of the cement gland at tailbud stages (as revealed by *Xanf1* and *Xag1* expression, respectively; Fig. 6I, J). This was correlated with the expansion of the SHA *Xanf1* domain (Fig. 6F) and with the up-regulation of *otx2* in the anterior ectoderm, presumably in the SHA cells (Fig. 6G), that we observed in some embryos at neural plate stages.

In conclusion, overexpression of *foxA4FL* produced a general posteriorisation in the head region, involving both the ectoderm and the neural ectoderm, with opposite changes in the expression of *otx2* and *Xanf1* (Fig. 10F). In the most severe phenotypes, the most cephalic structures, including the rostral forebrain and the cement gland, were lost. In milder phenotypes, we could appreciate an expansion of the caudal forebrain and of the SHA at the expense of the rostral forebrain and the cement gland.

In order to verify the specificity of the anteriorisation phenotype produced by FoxA4MO, we performed rescue experiments by co-injecting embryos with FoxA4MO + *foxA4CDS* mRNA, followed by analysis of *Xanf1/en2* (Fig. S5) and *otx2/krox20* expression (Fig. S4) at the neurula stage. For comparison, some embryos were co-injected with FoxA4MO + *foxA4FL* mRNA (Fig. S4), since the overexpression effects of the latter are expected to be prevented by FoxA4MO.

Injection of *foxA4CDS* mRNA produced similar results as overexpression of *foxA4FL* mRNA for both pair of markers analyzed (*Xanf1/en2*: Fig. S5C, G; *otx2/krox20*: Fig. S4C). While 20 ng of FoxA4MO produced the typical up-regulation and expansion of *Xanf1* with a concomitant down-regulation of *en2* in the majority of the injected embryos (70%, Fig. S5B, F, I), co-injection of 0.5 ng of *foxA4CDS* mRNA significantly prevented the FoxA4MO phenotype, which decreased to 18% of the injected embryos (Fig. S5I). Instead, the majority of the co-injected embryos (82%, Fig. S5I) were either similar to uninjected siblings (49%, Fig. S5H, I) or exhibited partial rescue (23% Fig. S5D, I) or overexpression phenotypes (10%; Fig. S5I). Co-injection of 1 ng of *foxA4CDS* mRNA completely reversed the FoxA4MO effect (100% of the co-injected embryos showed an overexpression phenotype; Fig. S5I). Therefore, *foxA4CDS* mRNA is able to rescue the effects of FoxA4MO on *Xanf1/en2*, indicating that the effects described for FoxA4 depletion on these anterior neural markers are specific.

While 20 ng of FoxA4MO produced the typical down-regulation of *otx2* in the majority of the injected embryos (76%, Fig. S4B, G), co-injection of 0.5 ng of *foxA4CDS* mRNA completely prevented the FoxA4MO phenotype (Fig. S4E, G). Instead, all the co-injected embryos (100%, Fig. S4G) were either similar to uninjected sibling controls (56%, Fig. S4E, G) or exhibited partial rescue (44%, Fig. S4G). As expected, co-injection of the same dose of *foxA4FL* mRNA was unable to rescue the FoxA4MO effect, showing the same proportion of the typical FoxA4MO effect on *otx2* (76%, Fig. S4F, G) as embryos injected with FoxA4MO alone. In addition, 20 ng of FoxA4MO notably increased the proportion of embryos without *krox20* expression in r5 as compared to control siblings (Fig. S4B, H). Remarkably, co-injection of 0.5 ng of *foxA4CDS* mRNA restored the expression of *krox20* in r5 to normal proportions at this stage (Fig. S4E, H), whereas the same dose of co-injected *foxA4FL* mRNA was unable to do so (Fig. S4F, H). Therefore, *foxA4CDS* mRNA (but not *foxA4FL* mRNA) was able to rescue the effects of FoxA4MO on *otx2/krox20*, indicating that the effects described for FoxA4 depletion on these anterior neural markers are specific.

### Depletion of FoxA4 leads to head defects at later stages and increases apoptosis

At tailbud stages, FoxA4 morphants showed reduced heads with smaller eyes (Fig. 2M, Q, O, S; Fig. 8H–L). In embryos that were unilaterally depleted from FoxA4, the telencephalic-caudal diencephalic gap of *otx2* expression disappeared, resulting in a fusion of the rostral and caudal subdomains (red arrowhead, Fig. 8G). The eye was severely reduced or absent on the injected side.

Next, we analysed whether the expression of other forebrain markers were changed in FoxA4 morphants. At tailbud stage, *six3* transcripts are mainly distributed in the eyes and in the rostral-ventral diencephalon [86] (Fig. 8C, F), while *emx1* is expressed in two bilateral stripes in the dorsal telencephalon, flanking the *otx2* telencephalic domain [87] (Fig. 8D, F). In uniformly depleted FoxA4 tailbuds, *six3* and *emx1* expression virtually disappeared. *Six3* was reduced to a small point and the *emx1* stripes were almost fused in the midline (Fig. 8I, J, L). The whole domain of *otx2* was reduced and its bilateral telencephalic subdomain was fused in the midline and fused with the caudal diencephalic subdomain. The eyes were smaller and less separated than in CtrlMO-injected siblings (Fig. 8H, L). Therefore, although at neural plate stage, FoxA4 morphants showed a posterior expansion of the presumptive rostral forebrain, at tailbud stages, the expression domains of rostral forebrain markers was reduced.

**Figure 11:**
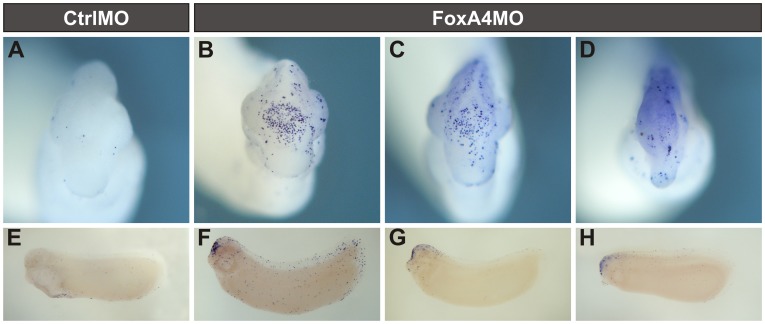
FoxA4 depletion induced robust cell death in the cephalic region. TUNEL analysis of stage 28 embryos injected with 20 ng of CtrlMO or FoxA4MO prior to the first cleavage. (A–D) Anterior views. (E–H) Lateral views of the same embryos shown in A–D, cleared in Murray's solution. CtrlMO-injected embryos showed scattered apoptotic cells (blue dots) (A, E). FoxA4MO-injected siblings presented accumulation of apoptotic cells in the head region (42%, n = 12) (B–D, F–H).

DML development was drastically affected in FoxA4 morphants. This signalling centre is the source of morphogens such as *shh*, required for the maintenance of the CNS along the A-P axis and for the subdivision of the forebrain in order to develop separate eyes and the two telencephalic hemispheres [1] [3] [88] [89]. Therefore, we wondered whether an increase in cell death might contribute to the reduction of the head. TUNEL analysis revealed that FoxA4MO induced massive cephalic apoptosis (Fig. 11).

In conclusion, rostral forebrain specification was favoured at neural plate stage in FoxA4 morphants, but at tailbud stages, the brain failed to reach its normal size and bilateral separation. One possible explanation is a decrease in signals from the DML. In addition, the caudal shift of the anterior border of the neural plate might also contribute to the reduction in the brain size.

### FoxA4 morphants show an abnormal A–P and D–V pattern of neurogenesis along the neural tube

We wondered whether neurogenesis was affected after FoxA4 depletion. FoxA4 morphant larvae showed differential changes in the expression of *N-tubulin* along the A–P axis. *N-tubulin* was down-regulated in the anterior region, including the hindbrain (white bar, Fig. 9B), while in the spinal cord *N-tubulin* expression increased and its pattern revealed projections arising from the CNS that were not observed on the non-injected side or in CtrlMO-injected siblings (Fig. 9A–D, red arrow). In the head, development of the trigeminal and vestibulocochlear nerves (Fig. 9E) [90] was impaired (red arrowhead, Fig. 9H). These results indicate that FoxA4 differentially influences head and trunk neurogenesis.

Transversal sections revealed that the increase of *N-tubulin* in the trunk occurred on the dorsal neural tube, corresponding to Rohon Beard sensory neurons, from where the projections are originated (yellow arrow, Fig. 9M). Supporting this, overexpression of *foxA4* leads to suppression of these neurons [31]. Fewer nuclei were present on the ventral neural tube on the injected side (compare light blue arrows, Fig. 9M, N). Notochord cells were absent or disorganised (green arrow, Fig. 9M, N). These results suggest that FoxA4 depletion favours dorsal fates and disfavours ventral development in the neural tube, probably because of DML impairment.

### *FoxA4* expression in the BCNE is directly involved in the A–P regionalisation of the anterior neural plate

It was previously described that the CNS can develop in *Xenopus* when mesoderm induction is blocked with the Nodal antagonist Cerberus-short (Cer-S). These mesodermless embryos still form brain tissue that express anterior neural markers (e.g. the caudal forebrain regulator *otx2* and the midbrain/hindbrain boundary marker en2, among others) and develop a cyclopic eye [37] [91]. However, it has not been tested before whether the rostral forebrain regulator *Xanf1* can be induced in the absence of mesoderm. In addition, Cer-S was also a useful tool to demonstrate that *chd* expression in the BCNE depends on β-catenin but not on Nodal signalling, and later, at gastrulation, it becomes dependent on Nodal [91].

**Figure 12:**
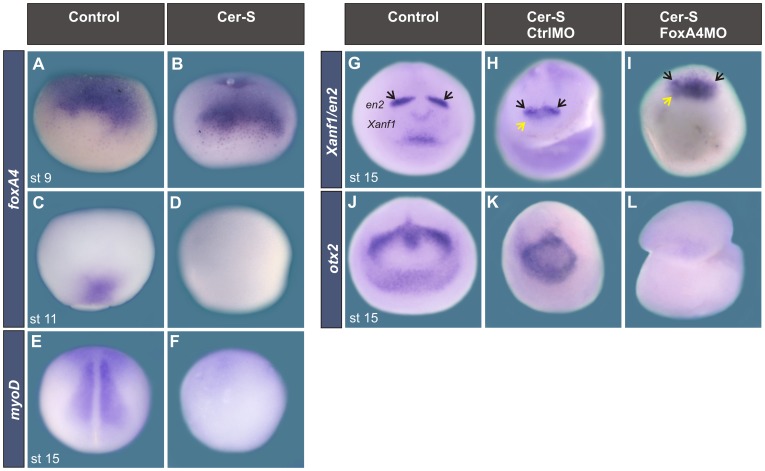
FoxA4MO reproduced the effects on anterior neural markers in embryos in which mesoderm induction was blocked with Cer-S. (**A–D**) Expression of *foxA4* in sibling controls (A, C) or in embryos injected with Cer-S mRNA (B, D), analysed at stage 9 (A, B) or at stage 11 (C, D). After blocking mesoderm induction, *foxa4* expression persisted in the BCNE (B) (100%, n = 15) but was suppressed from the axial mesoderm (D) (100%, n = 7). (**E, F**) *MyoD* expression at neural plate stage in a sibling control (E) and in an embryo injected with Cer-S. Mesoderm was suppressed in 100% of the injected embryos analyzed with *myoD* (100%, n = 8). (**G–I**) Expression of *Xanf1* and *en2* at neural plate stage in a sibling control (G), and in embryos in which mesoderm induction was blocked with Cer-S and were injected at the 1-cell stage with either 20 ng of CtrlMO (H) or 20 ng of FoxA4MO (I). Embryos injected with CtrlMO + Cer-S showed expression of *Xanf1* (yellow arrow) and *en2* (black arrow) (H, 100%, n = 8). In embryos injected with FoxA4MO + Cer-S, *Xanf1* was expanded and up-regulated (yellow arrow) and *en2* was down-regulated (black arrow) (I, 100%, n = 10) in comparison to embryos injected with CtrlMO + Cer-S (H). (J–L) Expression of *otx2* at neural plate stage in a sibling control (J), and in embryos in which mesoderm induction was blocked with Cer-S and were injected at the 1-cell stage with either 20 ng of Ctrl MO (K) or 20 ng of FoxA4MO (L). Embryos injected with CtrlMO + Cer-S showed expression of *otx2* (K, 100%, n = 4). In embryos injected with FoxA4MO + Cer-S, *otx2* was down-regulated (L, 100%, n = 20) in comparison to embryos injected with CtrlMO + Cer-S (K).

To study whether the A-P changes that we observed in the anterior neural plate can be ascribed to depletion of FoxA4 from the BCNE or from the axial mesoderm (DML), we made use of Cer-S., We first addressed whether Cer-S injection could affect *foxA4* expression in the BCNE or during gastrulation. Embryos injected with Cer-S mRNA and analysed at stage 9 and stage 11 clearly revealed that after blocking Nodal signalling and mesoderm induction, *foxA4* expression persisted in the BCNE (Fig. 12A, B) but was abolished in gastrulae (Fig. 12C, D). This is similar to the behaviour of *chd* [91].

Next, we addressed if *foxA4* expression in the BCNE is required for A–P patterning in the anterior neural plate. To answer this question, we analysed whether *foxA4* knock-down could reproduce the effects on *Xanf1, en2* and *otx2* that we described in Fig. 7 in embryos in which mesoderm induction was blocked with Cer-S. Thus, we compared the expression of these anterior neural markers between embryos injected with Cer-S (in which *foxA4* expression persists in the BCNE but it is suppressed from the axial mesoderm, which does not develop) and embryos injected with Cer-S + FoxA4MO. The absence of mesoderm was confirmed by suppression of *myoD* expression (Fig. 12E, F). In mesodermless embryos, although *Xanf1*, *en2* and *otx2* were expressed in the neural ectoderm, they showed smaller expression domains than in sibling controls (compare Fig. 12H, K with G, J). This indicates that although brain specification (including the rostral forebrain marker *Xanf1*) can occur in the absence of mesoderm, mesoderm-derived signals are necessary for maintaining a normal brain size and pattern. Notwithstanding this fact, it was remarkable that in mesodermless embryos depleted from FoxA4, *Xanf1* was up-regulated, while *en2* and *otx2* were down-regulated in relation to mesodermless embryos that were not injected with FoxA4MO (compare Fig. 12I, L with H, K). Therefore, in embryos lacking mesoderm we could still observe the same behaviour of the anterior neural markers as in embryos in which mesoderm induction was not blocked. Since in Cer-S injected embryos, *foxA4* expression only remains in the BCNE but it is absent from the mesoderm, these results indicate that *foxA4* expression in the BCNE is directly involved in the A–P regionalisation of the anterior neural plate, and it is necessary to restrict rostral forebrain specification.

**Figure 13:**
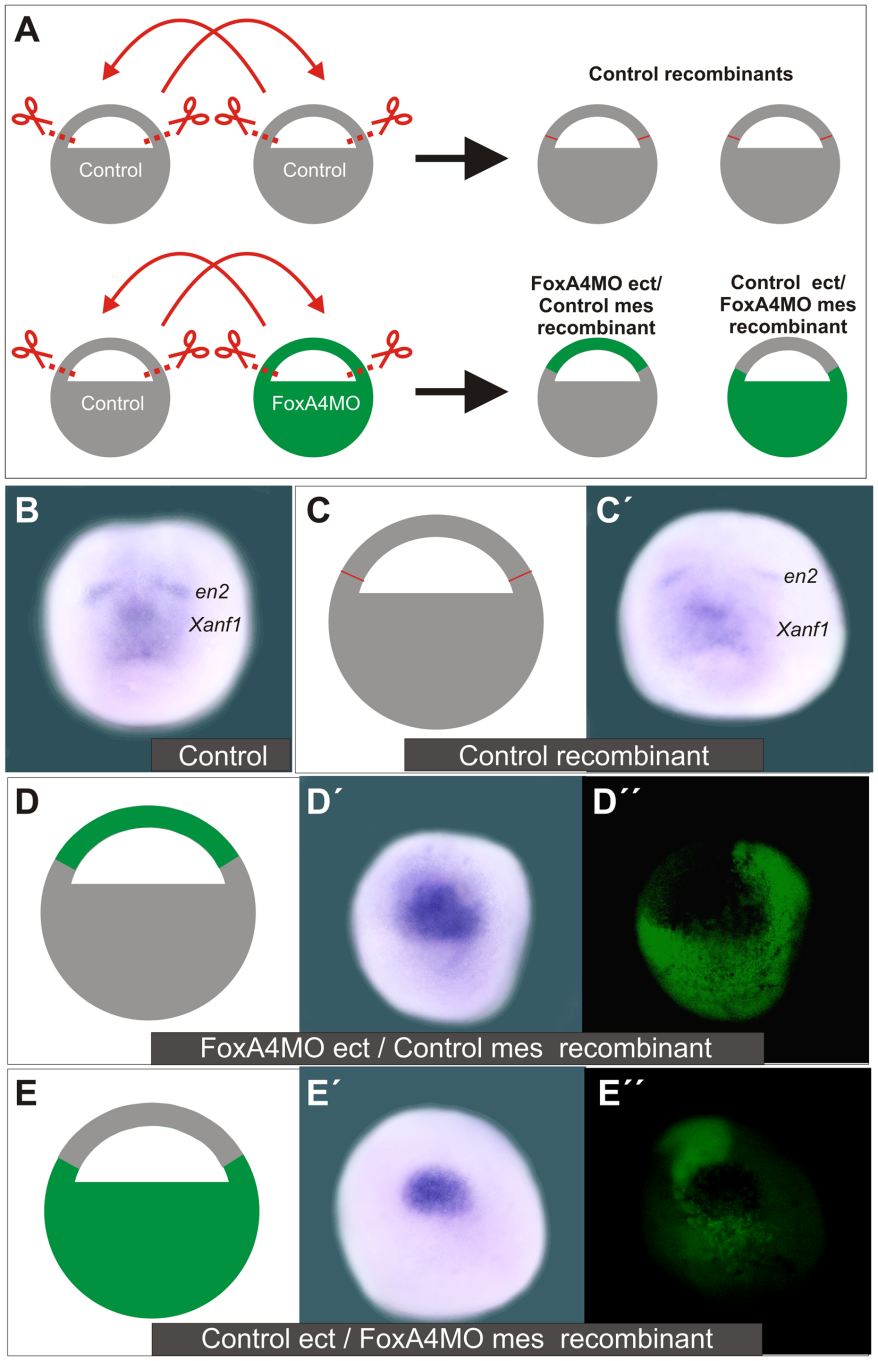
Depletion of FoxA4 from the ectoderm expands *Xanf1* and suppresses *en2* in the anterior neural plate. (**A**) Embryos were injected at the 1-cell stage with 20 ng of FoxA4MO +20 ng of DOG (green) or were left uninjected (grey). At stage 9, ectodermal explants were excised and the following recombinants were obtained: in Control recombinants, the ectodermal explant of a recipient uninjected embryo was replaced with the ectodermal explant from an uninjected donor. In FoxA4MO ect/Control mes recombinant, the ectoderm was provided by a FoxA4MO-injected donor (green), while the mesoderm derived from an uninjected sibling recipient (grey). In Control ect/FoxA4MO mes recombinant, the ectoderm was provided by an uninjected sibling donor (grey), while the mesoderm derived from a FoxA4MO-injected recipient (green). (**B–E″**) Analysis of *Xanf1* and *en2* when uninjected sibling controls (B) reached neurula stage. (C,C′) Control recombinant. (D–D″) FoxA4MO ect/Control mes recombinant. (E–E″) Control ect/FoxA4MO mes recombinant. (D″ and E″) DOG fluorescence of the images shown in D′, E′, respectively.

To corroborate this, we analysed the pattern of *Xanf1* and *en2* in recombinants in which the ectoderm was provided by a FoxA4MO-injected donor, while the mesoderm derived from an uninjected sibling recipient (Fig. 13A, FoxA4MO ect/Control mes recombinant), and in recombinants in which the ectoderm was provided by an uninjected sibling donor, while the mesoderm derived from a FoxA4MO-injected recipient (Fig. 13A, Control ect/FoxA4MO mes recombinant). Their patterns were compared to those of uninjected controls (Fig. 13B) and of control recombinants between uninjected embryos (Fig. 13A, C–C′). FoxA4MO ect/Control mes recombinants showed an up-regulation and expansion of the *Xanf1* domain in the anterior neural plate while the *en2* stripes were not apparent (100%, n = 7; Fig. 13D–D″). This result is similar to the effect that we observed in intact embryos injected with FoxA4MO, supporting the observations obtained from the experiments with mesodermless embryos. On the other hand, Control ect/FoxA4MO recombinants did not show a normal *Xanf1/en2* domain in the anterior neural plate. While *en2* expression was suppressed, *Xanf1* expression was not widely expanded through the anterior neural plate as in FoxA4MO ect/Control mes recombinants, and the area normally demarcated by this marker appeared to be restricted to a smaller area on the DML, filled with *Xanf1* transcripts (100%, n = 9; Fig. 13E–E″).

In overall, these results together indicate that *foxA4* is directly necessary in the BCNE/ectodermal brain precursors in the blastula to restrict rostral forebrain specification. However, signals from the mesoderm and an intact function of *foxA4* in the axial mesoderm are required to maintain the normal size and pattern of the prospective brain at neural plate stages.

## Discussion

**Figure 14:**
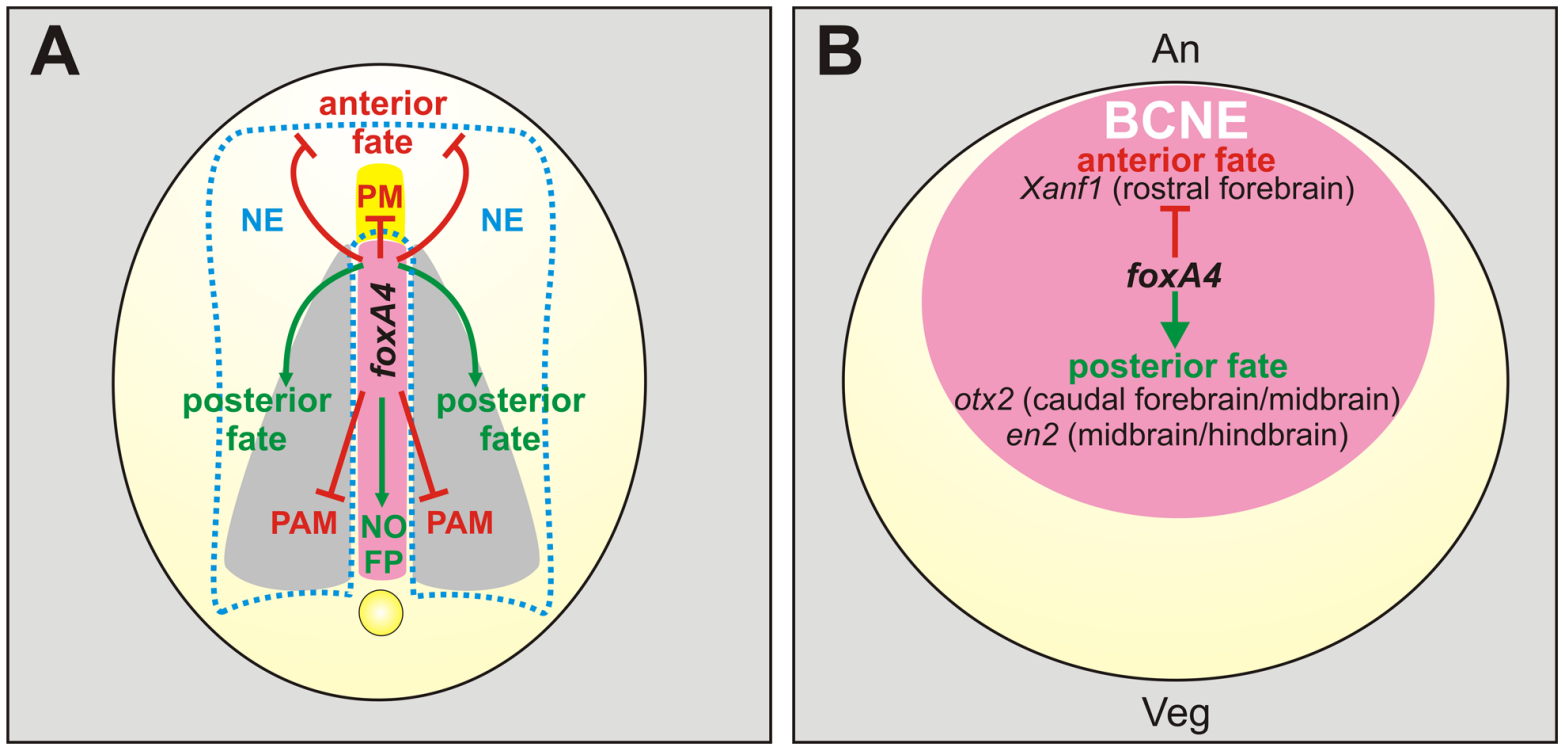
Model for *foxA4* function in *Xenopus* embryos. (**A**) Summary of *foxA4* functions in A–P regionalisation and in DML development. For simplicity, they are depicted in a diagram of an embryo at neural plate stage, shown in dorsal view, when *foxA4* is only expressed in the DML (pink). *FoxA4* modulates A–P development by inhibiting anterior fates (red lines) in the axial mesoderm (prechordal mesoderm, PM) and in the neuroectoderm (NE) and ectoderm, while favouring posterior fates (green arrows) in the neural plate and the dorsal midline (notochord, NO; floor plate, FP). In the trunk, *foxA4* prevents the respecification of dorsal midline precursors towards contiguous fates, by inhibiting (red lines) the paraxial mesodermal fate (PAM). Yellow, prechordal mesoderm. Grey, paraxial mesoderm. The dotted light blue line demarcates de neural plate. (**B**) Diagram of a blastula stage embryo in dorsal view, showing the expression of *foxA4* in the BCNE centre (pink), which is composed by precursors of the Spemann's organiser and of the whole forebrain and most of the midbrain and hindbrain [37]. An, animal; Veg, vegetal. *FoxA4* modulates the initial CNS regionalisation by operating on the BCNE, favouring posterior fates among BCNE derivatives (green arrow), while restricting anterior fates (red line), as revealed by the markers *Xanf1* (rostral forebrain), *otx2* (caudal forebrain/midbrain), *en2* (midbrain/hindbrain boundary).

In the present work, we present evidence that *foxA4* plays an important role in the formation of the DML and in A–P patterning in *Xenopus laevis*. *FoxA4* is necessary for the specification and correct allocation of the components of the trunk DML and for the correct formation of the rostral forebrain and anterior ectodermal derivatives (Fig. 10). In overall, *foxA4* modulates A–P development, restricting anterior axial mesodermal and neural/ectodermal fates (Fig. 14).

### *FoxA4* is necessary for the development of the DML

The inhibition of *foxA4* resulted in loss of notochord and FP cells, which was accompanied by a posterior expansion of the PM and by the invasion of the midline by the paraxial mesoderm and the neuroectoderm (Fig. 3; Fig. 10A, B). Since there was not an increase in cell death, FoxA4 depletion most likely produced a respecification of DML precursors towards contiguous fates, favouring prechordal and paraxial mesoderm. Although some cells expressing *chd* ultimately remained at the level of notochordal gaps, they were found within the ventral neural tube and the dorsal endoderm, possibly occupying the place of the FP and the hypochord. Therefore, *foxA4* is normally required for the specification and organisation of the DML structures.

Although the combined domains of *foxA2* and *foxA4* throughout *Xenopus* development reproduce the *foxA2* profile of mammals [32], the early expression of *foxA4* during DML specification more closely resembles that of mammalian *foxa2*. It was suggested that the early functions of mouse *foxa2* are carried out by *foxA4* in frogs [32]. While *foxA2* knock-out completely abolished the development of the notochord and the FP in mice [24] [27], we found that the DML was partially ablated and severely disorganised after *foxA4* knock-down. This indicates that some important DML functions of mammalian *foxa2* are exerted by *foxA4* in *Xenopus*. The partial impairment is consistent with the observation that the expression of the axial marker *chd* could not be completely abolished in the organiser in FoxA4 morphants.

In zebrafish, inactivation of *foxA2* resulted in discontinuous FP development, but the notochord and the hypochord were not affected, indicating that this gene is required for the maintenance of regulatory genes in the fish FP [92]. Strikingly, combined knock-down of *foxA2* and *foxA3* produced localised disruptions of the three DML structures, and changed the fate of their precursors to paraxial and ventral neural tube specifications [93], resembling our DML phenotypes in FoxA4 morphants. Since a *foxA3* orthologue was never found in *Xenopus*, and *foxA4* genes were only isolated from amphibians, but not from fishes and higher vertebrates [21], *foxA4* might integrate the fish foxA2/A3 function. In conclusion, in lower vertebrates, members of the FoxA subfamily, including amphibian *foxA4*, are necessary for DML specification and allocation, restricting the paraxial and PM fates in order to prevent the invasion of the DML by contiguous tissues (Fig. 10A). This mode of action of FoxA members could be uncovered in lower vertebrates since the DML phenotypes were milder than those obtained in *foxa2* null mice embryos. The role of *foxA* genes in DML development might be related with the modulation of morphogenetic movements, perhaps favouring those typical of the chordamesoderm, since FoxA4 depletion inhibits axis elongation and produces complementary changes in *bra* and *gsc*, two genes that oppositely control convergent extension and active cell migration behaviours in the axial mesoderm.

### *FoxA4* inhibits anterior development

At neural plate stage, the attenuation of *foxA4* produced a general anteriorisation, involving both the ectoderm and the neural ectoderm, with complementary changes in the expression of *otx2* and *Xanf1*. The *Xanf1* domain corresponding to the ANR was expanded at the expense of the prospective caudal diencephalic/mesencephalic *otx2* subdomain, indicating an expansion of the presumptive rostral forebrain at the expense of the presumptive caudal forebrain and midbrain. In the ectodermal territory, the CGA shifted posteriorly at the expense of the SHA and the neural plate border (Fig. 10D, E). FoxA4 knock-down also affected the hindbrain, producing caudal shifts and inhibiting the midbrain/hindbrain boundary marker *en2*, and the rhombomeric markers *krox20* and *hoxb1* (Fig. 7). In the presumptive spinal cord, FoxA4 depletion shifted the anterior limit of *hoxb7* posteriorly (Fig. 7). In overall, our results indicate that knock-down of *foxA4* promotes anterior neural and ectodermal specification while disfavours posterior neural development (Fig. 14A). However, at tailbud stages, the brain collapsed, indicating that further maintenance of the anterior structures could not be sustained in FoxA4 morphants. The decrease of antiapoptotic signals from the disrupted DML might explain the reduction of the brain size in larvae. Interestingly, experiments with mesodermless embryos (injections of Cer-S alone; Fig. 12H, K) and with recombinants in which FoxA4 was depleted from the mesoderm (Fig. 13E–E′) indicate that signals from the mesoderm and an intact function of *foxA4* in the axial mesoderm are required to maintain the normal size and pattern of the prospective brain at neural plate stages.

Overexpression of *foxA4* produced a general posteriorisation in the head region at neural plate stage, with complementary changes in the neural and ectodermal subdomains of *otx2* and *Xanf1*, opposite to the ones observed after FoxA4 depletion at the same stage (Fig. 10D–F). Later, in the most affected larvae, the head was truncated and the rostral forebrain and the cement gland were lost. The milder phenotypes showed an expansion of the caudal forebrain at the expense of the rostral forebrain, with an expansion of the pituitary anlage (Fig. 6).

In conclusion, our experimental data demonstrate that *foxA4* is required for the establishment of the normal A–P patterning of the neural plate. We propose that *foxA4* inhibits rostral forebrain and anterior ectodermal development in favour of more posterior fates during early specification. Consistent with this, it was reported that overexpression of *foxA4* reduces the size of forebrain structures and increases the amount of posterior neural tissue [31]. Moreover, we show that *foxA4* is required for the correct segregation of the anterior ectodermal anlagen and the ANR.

It was proposed that *foxA4* controls the posterior medial limit of *Xanf1*, thus restricting its caudal expansion within the DML [36]. The same morpholino that we used here was employed by these authors, but it was injected at later cleavage stages, directed to the most animal blastomeres. In our study, the morpholino was more widely delivered, and we observed a significantly broader increase in the expression of *Xanf1*, compromising the whole anterior neural plate. Our findings indicate that *foxA4* confines the entire posterior limit of *Xanf1* within the anterior neural plate and is required to constrain the prospective rostral forebrain territory during neural specification. Consistent with this, the injection of *foxA4-VP16* mRNA to reverse the repressor function of *foxA4* resulted in an expansion of *Xanf1* lateral to the DML, almost reaching the *en2* stripe [36]. However, these authors only discussed the results obtained with their knock-down approach, which were restricted to the DML, as they were expected from the normal expression of *foxA4* in the FP. At the time of the study, the earlier expression of *foxA4* in the BCNE had not been reported.

The fates of the dorsal animal blastomeres are significantly mixed in terms of brain or spinal cord fates [94]. Moreover, the BCNE centre is composed by precursors of the whole forebrain and most of the midbrain and hindbrain and of the Spemann's organiser [37]. The induction of the anterior CNS starts at blastula stage and requires the combined activity of the BCNE and Nieuwkoop centres [37] [38] [95]. We have found that during early *Xenopus* development, Notch restricts the formation of the BCNE and controls the A–P regionalisation of the neural plate, suggesting that this pathway favours more posterior values among BCNE derivatives [96]. The similarity of the neural phenotypes obtained after manipulating Notch signalling or *foxA4* activity suggested that the latter might modulate the initial CNS regionalisation by operating on the BCNE. By knocking-down *foxA4* in mesodermless embryos, we could investigate the relevance of the expression of *foxA4* in the BCNE, independently of its expression in the mesoderm, demonstrating a direct impact of the early presence of this transcription factor on the initial A–P regionalisation in the anterior neural plate. In the absence of mesoderm, FoxA4MO still promoted the expansion of the rostral forebrain marker *Xanf1* and the inhibition of more posterior markers such as the caudal forebrain regulator *otx2* and the midbrain/hindbrain marker *en2* (Fig. 14B). Experiments with recombinant embryos in which the ectoderm derived from a FoxA4-depleted donor confirmed that *foxA4* is directly required in the ectodermal brain precursors in the blastula to restrict rostral forebrain specification. A direct regulation of *Xanf1* by *foxA4* in the neural ectoderm is supported by a previous study which reported the finding of cis-regulatory binding sites for *foxA4* in a 14 bp element of the *Xanf1* promoter which is critical for restricting its expression in the forebrain [36]. However, that study only paid attention to the DML aspect of *Xanf1* regulation by *foxA4*, and dismissed a putative role of *foxA4* in restricting *Xanf1* in the whole forebrain, as discussed above.

A rostral forebrain with telencephalic and rostral diencephalic compartments is a distinctive innovation of vertebrates [70] [97]. The chordate amphioxus, the closest living relative of the vertebrates, presents a forebrain with diencephalic characteristics. It was proposed that the emergence of *anf* genes in vertebrates ancestors allowed the development of the telencephalon and the rostral diencephalon by inhibiting caudal diencephalic and mesencephalic programmes executed by *otx2* and *pax6* genes [70]. Our results further indicate that the expression of *Xanf1* must be counterbalanced by *foxA4* in order to constrain the extension of the prospective rostral forebrain during the specification of A–P compartments in amphibians. Moreover, the latter appears to be necessary and sufficient to exert this role, since overexpression produced results opposite to those of the knock-down analysis. It will be interesting to elucidate whether other members of the FoxA subfamily play similar roles in the vertebrate lineage.

Apart from the defects in the D–V pattern of the neural tube, *foxa2* mutant mice present forebrain truncations [24] [27]. In addition to the expression in the node and derivatives, *foxa2* is also expressed in the anterior visceral endoderm (AVE), an extraembryonic signalling centre critical for head formation. Selective inactivation of the *foxa2* function in either the AVE or the DML revealed that the activity of this gene is required in both centres to specify the forebrain fate in mammals [25] [26] [28]. Moreover, expression of the *anf* gene was not detected or was very reduced at early stages in these conditional mutants. This resembles the situation in our recombinants in which FoxA4 was only depleted from the endomesoderm, resulting in a reduction of the *Xanf1* domain in comparison to the area delimited by this transcription factor in sibling controls or control transplants. This suggests that amphibian *foxA4* function in the endomesoderm is also necessary for head development.

### A dual function of FoxA4?

Early studies proposed that FoxA4 is a transcriptional activator [34] [35]. On the other hand, FoxA4 is able to bind two elements in the promoter of *Xanf1*, which are essential for the restriction of its expression to the anterior region of the CNS. This, together with the results obtained with the *foxA4-VP16* construct, indicates that FoxA4 acts as a transcriptional repressor of *Xanf1* [36]. We observed that the knock-down of *foxA4* produced opposite effects on the expression of genes in the anterior and posterior regions of the embryo. FoxA4 morphants showed a repression of posterior markers such as *hoxb7* and *hoxc6* and of DML markers in the trunk, and an up-regulation of anterior markers like *gsc, frzb1* and *Xanf1*. Therefore, the possibility that FoxA4 acts as activator or repressor depending on the A–P environment should be considered in the future.

## Supporting Information

Figure S1 **Rescue experiments showing that FoxA4MO effects on DML and paraxial mesoderm markers are specific.** (**A–E, K**) Expression of *chd*, analyzed at neural plate stage. (**F–J, L**) Expression of *myoD*, analyzed at neural plate stage. (A,F) Sibling controls. Embryos were injected at the 1-cell stage with 20 ng of FoxA4MO (B, G), 0.5 ng of *FoxA4CDS* mRNA (C, H), or 20 ng of FoxA4MO +0.5 ng of *FoxA4CDS* mRNA (D, E, I, J). In (K, L), the bars compare the percentage of embryos showing the indicated phenotypes between injections of FoxA4MO alone, *FoxA4CDS* mRNA alone or FoxA4MO + *FoxA4CDS* mRNA, as follows: similar to control (green), FoxA4MO phenotype (blue), overexpression phenotype (red), partial rescue (light green). The total number of injected embryos is indicated below each bar (n). Numbers inside the bars indicate the percentage of embryos exhibiting the corresponding phenotype.
(TIF)

Figure S2 ***FoxA4*** **inhibition impaired notochord development.** Transverse sections through the trunk level of stage 26 embryos injected into 2 dorsal cells at the 4-cell stage with 20 ng of CtrlMO (A–B′) or FoxA4MO (C–F′), and hybridised with a *chd* probe. (A′–F′) Hoescht staining. At this stage, *chd* normally has a strong expression in the notochord (no), but some transcripts are also found in the floor plate (fp). In FoxA4 morphants the notochord was disorganised and did not segregate from the FP or the dorsal endoderm (C), or was absent and *chd* + cells were found in the ventral neural tube/FP (black arrowheads) or in the dorsal endoderm/hypochord (red arrowheads) (D,E,F). The somites (so) tended to fuse in the midline (F′) and the distribution of their nuclei suggest that they were also disorganised (compare C′, D′, E′, F′ with A′, B′).
(TIF)

Figure S3 **Apoptosis and proliferation were normal in FoxA4-depleted embryos.** Embryos were injected with 20 ng of CtrlMO (A, C, E) or FoxA4MO (B, D, F) before the first cleavage and were processed for TUNEL (A–D) or for immunohistochemistry of Phosphohistone H3 (PH-3) (E, F). Embryos injected with FoxA4MO (n = 22) presented similar levels of apoptosis with respect to CtrlMO-injected siblings (n = 21) at stage 15 (A, B) or 22 (C, D). Injection of FoxA4MO did not change the proliferation level at stage 15 (E–G). Embryos were cleared in Murray's solution. (G) Four FoxA4MO-injected embryos (red bar) and four CtrlMO-injected siblings (green bar) corresponding to the groups shown in E, F were sectioned in the sagittal plane. The number of PH-3 + nuclei and the area of each section were measured with Image-Pro Plus software in a total of 21 successive sections, one corresponding to the medial plane and ten of each side of the embryo. Results are represented as the ratio between the number of PH-3 + nuclei and the area of the sections (PH-3 nuclei/area). There were not significant changes in the number of proliferating cells between FoxA4MO- and CtrlMO-injected siblings. *P*<0.9272, two-tailed t-test.
(TIF)

Figure S4 **Rescue experiments showing that FoxA4MO effects on** ***otx2*** **and** ***krox20*** **are specific.** (**A–F**) Expression of *Xanf1* and *en2*. Embryos were fixed in more advanced neurula stages than before (stage 15/16) and we were able to analyse *krox20* expression in the 3^rd^ (r3) and 5^th^ (r5) presumptive rhombomeres. (A) Sibling control. Embryos were injected at the 1-cell stage (B–F) with 20 ng of FoxA4MO (B), 0.5 ng of *FoxA4CDS* mRNA (C), 0.5 ng of *FoxA4FL* mRNA (D), 20 ng of FoxA4MO +0.5 ng of *FoxA4CDS* mRNA (E), or 20 ng of FoxA4MO +0.5 ng of *FoxA4FL* mRNA (F). (**G**) Analysis of the *otx2* phenotypes. The bars compare the percentage of embryos showing the indicated phenotypes between injections of FoxA4MO alone, *FoxA4CDS* mRNA alone, *FoxA4FL mRNA* alone, FoxA4MO + *FoxA4CDS* mRNA, or FoxA4MO + *FoxA4FL* mRNA as follows: similar to control (green), FoxA4MO phenotype (blue), overexpression phenotype (red), partial rescue (light green). The total number of injected embryos is indicated below each bar (n). Numbers inside the bars indicate the percentage of embryos exhibiting the corresponding phenotype. (**H**) Analysis of *krox20* expression in r5. Embryos were scored as showing (r5+, lilac) or not showing (r5-, orange) *krox20* expression in the presumptive r5 territory. The bars compare the percentage of embryos with or without *krox20* expression in r5 between injections of FoxA4MO alone, *FoxA4CDS* mRNA alone, *FoxA4FL* mRNA alone, FoxA4MO + *FoxA4CDS* mRNA, or FoxA4MO + *FoxA4FL* mRNA. The total number of injected embryos is indicated below each bar (n). Numbers inside the bars indicate the percentage of embryos exhibiting the corresponding phenotype.
(TIF)

Figure S5 **Rescue experiments showing that FoxA4MO effects on** ***Xanf1*** **and** ***en2*** **are specific.** (**A–I**) Expression of *Xanf1* and *en2*, analysed at neurula stage. (A,E) Sibling controls. Embryos were injected at the 1-cell stage (B–D) or at 1 dorsal blastomere at the 4-cell stage (F–H) with 20 ng of FoxA4MO (B, F, I), 0.5 ng (C, G, I) or 1 ng (I) of *FoxA4CDS* mRNA, 20 ng of FoxA4MO +0.5 ng of *FoxA4CDS* mRNA (D,H,I), or 20 ng of FoxA4MO +1 ng of *FoxA4CDS* mRNA (I). In (I), the bars compare the percentage of embryos showing the indicated phenotypes between injections of FoxA4MO alone, *FoxA4CDS* mRNA alone or FoxA4MO + FoxA4CDS mRNA, as follows: similar to control (green), FoxA4MO phenotype (blue), overexpression phenotype (red), partial rescue (light green). The total number of injected embryos is indicated below each bar (n). Numbers inside the bars indicate the percentage of embryos exhibiting the corresponding phenotype. Black arrowhead in (F), caudal shift and down regulation of *en2* on the injected side, typical of the FoxA4 morphant phenotype. Black arrow in (F), up-regulation and caudal expansion of the *Xanf1* domain on the injected side, typical of the FoxA4 morphant phenotype.
(TIF)
