# Supplementary figures and images for: FoxA4 Favours Notochord Formation by Inhibiting Contiguous Mesodermal Fates and Restricts Anterior Neural Development in Xenopus Embryos
Source: PLoS One. 2014 Oct 24;9(10):e110559. doi: 10.1371/journal.pone.0110559 (PMC4208771; doi:10.1371/journal.pone.0110559)

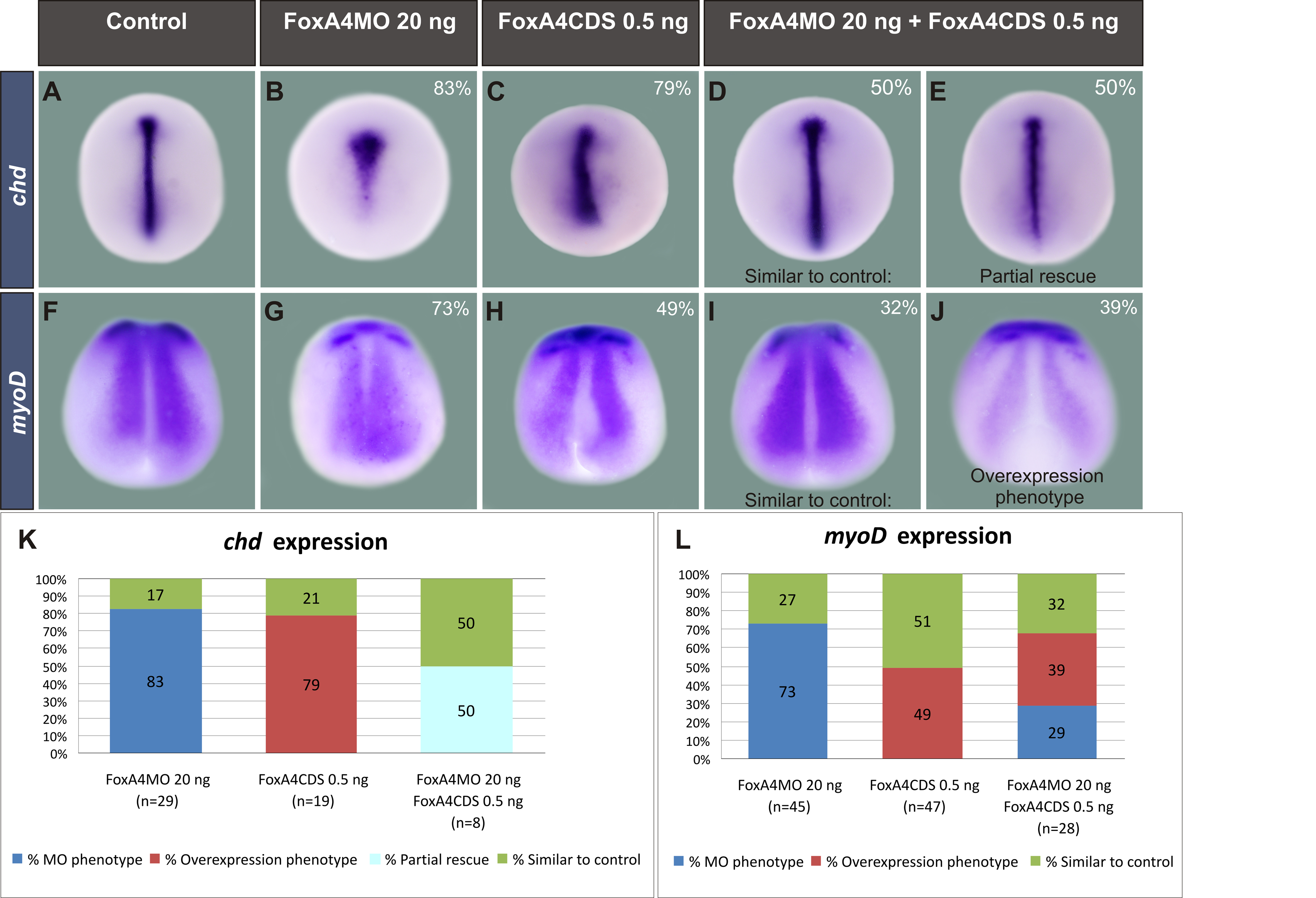

Supplement: Figure S1 — Rescue experiments showing that FoxA4MO effects on DML and paraxial mesoderm markers are specific. (A–E, K) Expression of chd, analyzed at neural plate stage. (F–J, L) Expression of myoD, analyzed at neural plate stage. (A,F) Sibling controls. Embryos were injected at the 1-cell stage with 20 ng of FoxA4MO (B, G), 0.5 ng of FoxA4CDS mRNA (C, H), or 20 ng of FoxA4MO +0.5 ng of FoxA4CDS mRNA (D, E, I, J). In (K, L), the bars compare the percentage of embryos showing the indicated phenotypes between injections of FoxA4MO alone, FoxA4CDS mRNA alone or FoxA4MO + FoxA4CDS mRNA, as follows: similar to control (green), FoxA4MO phenotype (blue), overexpression phenotype (red), partial rescue (light green). The total number of injected embryos is indicated below each bar (n). Numbers inside the bars indicate the percentage of embryos exhibiting the corresponding phenotype. (TIF) [file pone.0110559.s001.tif]

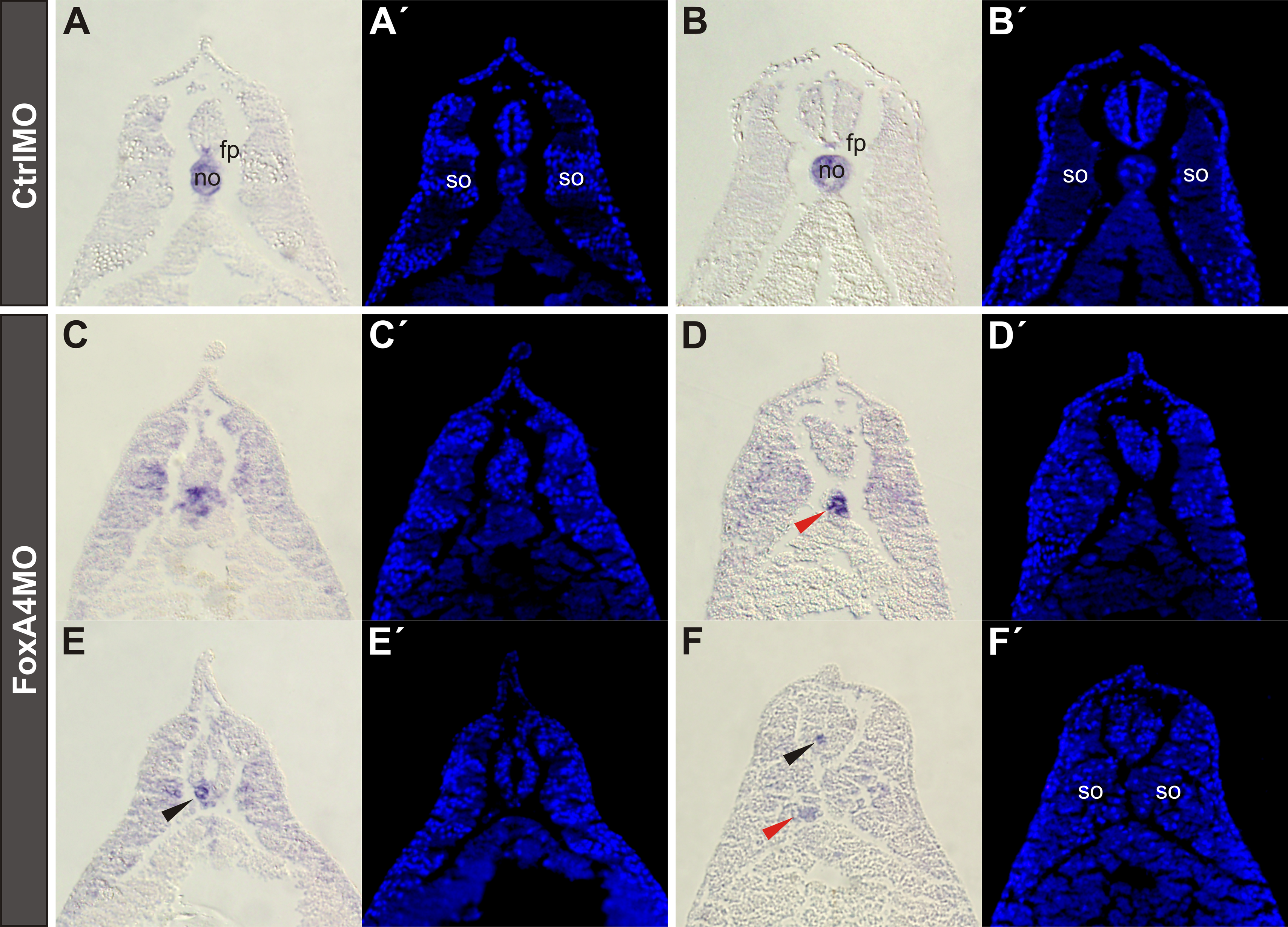

Supplement: Figure S2 — FoxA4 inhibition impaired notochord development. Transverse sections through the trunk level of stage 26 embryos injected into 2 dorsal cells at the 4-cell stage with 20 ng of CtrlMO (A–B′) or FoxA4MO (C–F′), and hybridised with a chd probe. (A′–F′) Hoescht staining. At this stage, chd normally has a strong expression in the notochord (no), but some transcripts are also found in the floor plate (fp). In FoxA4 morphants the notochord was disorganised and did not segregate from the FP or the dorsal endoderm (C), or was absent and chd + cells were found in the ventral neural tube/FP (black arrowheads) or in the dorsal endoderm/hypochord (red arrowheads) (D,E,F). The somites (so) tended to fuse in the midline (F′) and the distribution of their nuclei suggest that they were also disorganised (compare C′, D′, E′, F′ with A′, B′). (TIF) [file pone.0110559.s002.tif]

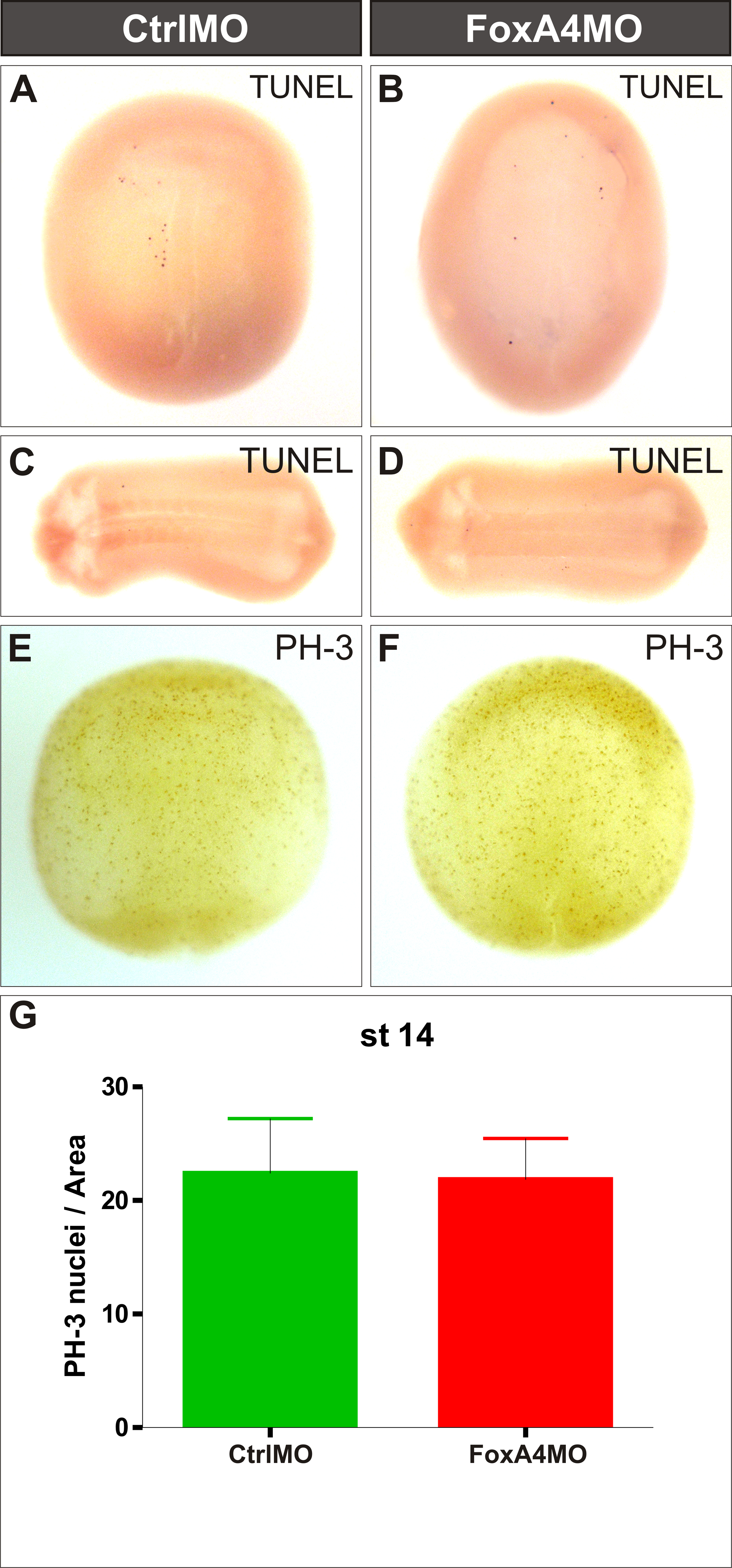

Supplement: Figure S3 — Apoptosis and proliferation were normal in FoxA4-depleted embryos. Embryos were injected with 20 ng of CtrlMO (A, C, E) or FoxA4MO (B, D, F) before the first cleavage and were processed for TUNEL (A–D) or for immunohistochemistry of Phosphohistone H3 (PH-3) (E, F). Embryos injected with FoxA4MO (n = 22) presented similar levels of apoptosis with respect to CtrlMO-injected siblings (n = 21) at stage 15 (A, B) or 22 (C, D). Injection of FoxA4MO did not change the proliferation level at stage 15 (E–G). Embryos were cleared in Murray's solution. (G) Four FoxA4MO-injected embryos (red bar) and four CtrlMO-injected siblings (green bar) corresponding to the groups shown in E, F were sectioned in the sagittal plane. The number of PH-3 + nuclei and the area of each section were measured with Image-Pro Plus software in a total of 21 successive sections, one corresponding to the medial plane and ten of each side of the embryo. Results are represented as the ratio between the number of PH-3 + nuclei and the area of the sections (PH-3 nuclei/area). There were not significant changes in the number of proliferating cells between FoxA4MO- and CtrlMO-injected siblings. P<0.9272, two-tailed t-test. (TIF) [file pone.0110559.s003.tif]

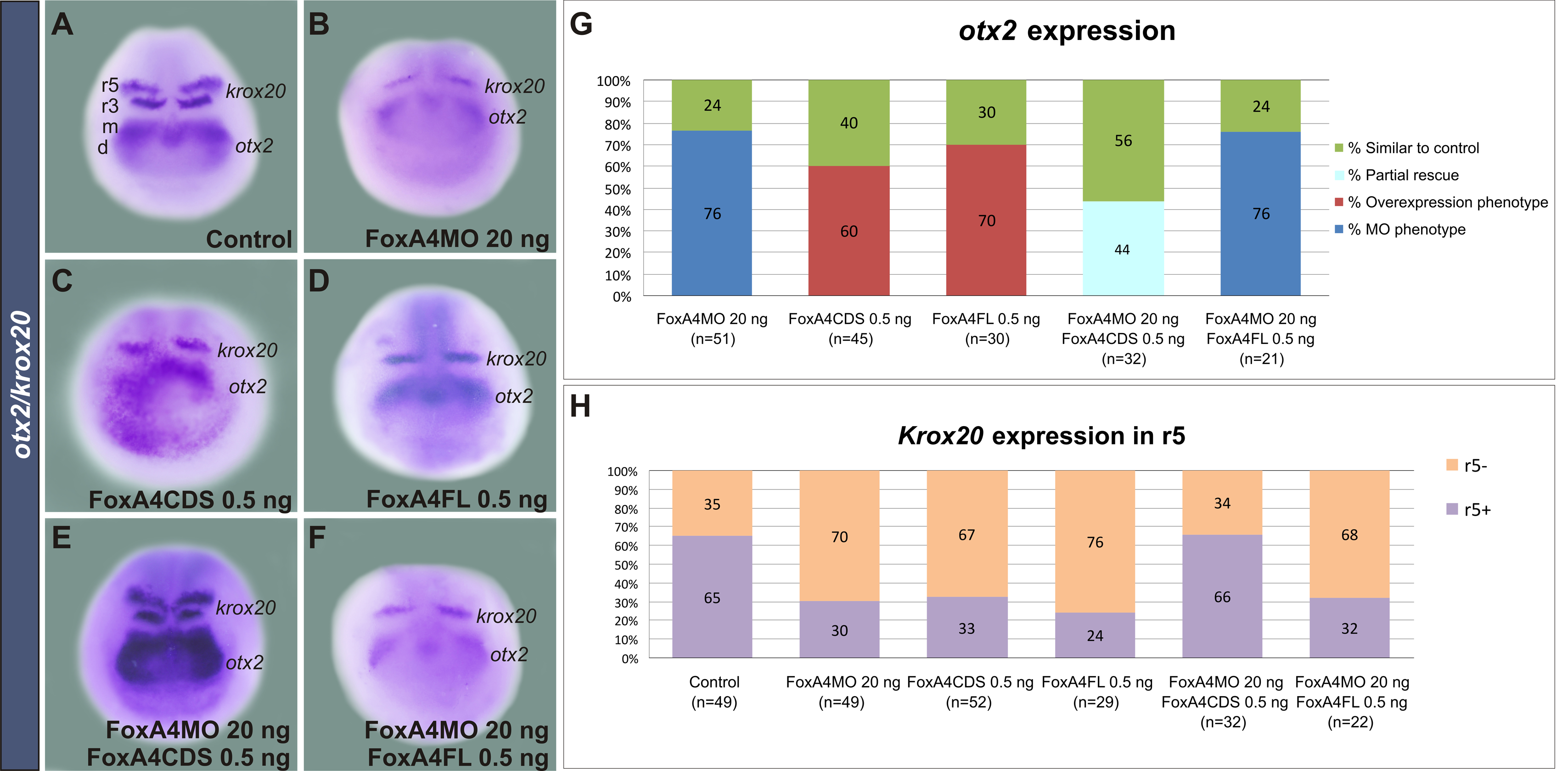

Supplement: Figure S4 — Rescue experiments showing that FoxA4MO effects on otx2 and krox20 are specific. (A–F) Expression of Xanf1 and en2. Embryos were fixed in more advanced neurula stages than before (stage 15/16) and we were able to analyse krox20 expression in the 3rd (r3) and 5th (r5) presumptive rhombomeres. (A) Sibling control. Embryos were injected at the 1-cell stage (B–F) with 20 ng of FoxA4MO (B), 0.5 ng of FoxA4CDS mRNA (C), 0.5 ng of FoxA4FL mRNA (D), 20 ng of FoxA4MO +0.5 ng of FoxA4CDS mRNA (E), or 20 ng of FoxA4MO +0.5 ng of FoxA4FL mRNA (F). (G) Analysis of the otx2 phenotypes. The bars compare the percentage of embryos showing the indicated phenotypes between injections of FoxA4MO alone, FoxA4CDS mRNA alone, FoxA4FL mRNA alone, FoxA4MO + FoxA4CDS mRNA, or FoxA4MO + FoxA4FL mRNA as follows: similar to control (green), FoxA4MO phenotype (blue), overexpression phenotype (red), partial rescue (light green). The total number of injected embryos is indicated below each bar (n). Numbers inside the bars indicate the percentage of embryos exhibiting the corresponding phenotype. (H) Analysis of krox20 expression in r5. Embryos were scored as showing (r5+, lilac) or not showing (r5-, orange) krox20 expression in the presumptive r5 territory. The bars compare the percentage of embryos with or without krox20 expression in r5 between injections of FoxA4MO alone, FoxA4CDS mRNA alone, FoxA4FL mRNA alone, FoxA4MO + FoxA4CDS mRNA, or FoxA4MO + FoxA4FL mRNA. The total number of injected embryos is indicated below each bar (n). Numbers inside the bars indicate the percentage of embryos exhibiting the corresponding phenotype. (TIF) [file pone.0110559.s004.tif]

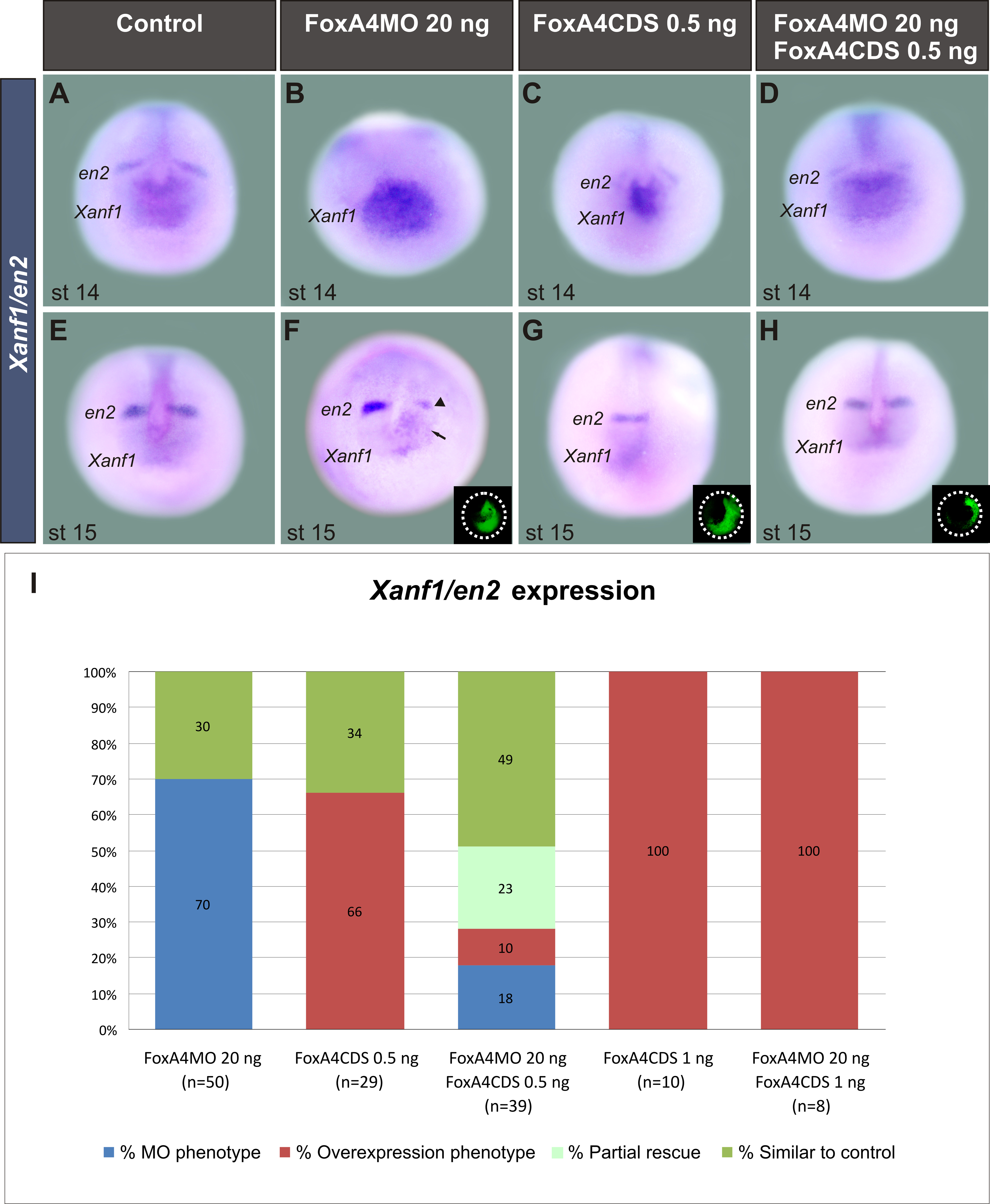

Supplement: Figure S5 — Rescue experiments showing that FoxA4MO effects on Xanf1 and en2 are specific. (A–I) Expression of Xanf1 and en2, analysed at neurula stage. (A,E) Sibling controls. Embryos were injected at the 1-cell stage (B–D) or at 1 dorsal blastomere at the 4-cell stage (F–H) with 20 ng of FoxA4MO (B, F, I), 0.5 ng (C, G, I) or 1 ng (I) of FoxA4CDS mRNA, 20 ng of FoxA4MO +0.5 ng of FoxA4CDS mRNA (D,H,I), or 20 ng of FoxA4MO +1 ng of FoxA4CDS mRNA (I). In (I), the bars compare the percentage of embryos showing the indicated phenotypes between injections of FoxA4MO alone, FoxA4CDS mRNA alone or FoxA4MO + FoxA4CDS mRNA, as follows: similar to control (green), FoxA4MO phenotype (blue), overexpression phenotype (red), partial rescue (light green). The total number of injected embryos is indicated below each bar (n). Numbers inside the bars indicate the percentage of embryos exhibiting the corresponding phenotype. Black arrowhead in (F), caudal shift and down regulation of en2 on the injected side, typical of the FoxA4 morphant phenotype. Black arrow in (F), up-regulation and caudal expansion of the Xanf1 domain on the injected side, typical of the FoxA4 morphant phenotype. (TIF) [file pone.0110559.s005.tif]
